# Redox Modifications of Proteins of the Mitochondrial Fusion and Fission Machinery

**DOI:** 10.3390/cells9040815

**Published:** 2020-03-27

**Authors:** Christina Wolf, Víctor López del Amo, Sabine Arndt, Diones Bueno, Stefan Tenzer, Eva-Maria Hanschmann, Carsten Berndt, Axel Methner

**Affiliations:** 1 Institute of Molecular Medicine, University Medical Center of the Johannes-Gutenberg University Mainz, 55131 Mainz, Germany; wolf.christina90@gmail.com (C.W.); diones.bueno@gmail.com (D.B.); 2 Section of Cell and Developmental Biology, University of California San Diego, La Jolla, CA 92093, USA; vlopezdelamo@ucsd.edu; 3 Institute for Immunology, University Medical Center of the Johannes-Gutenberg University Mainz, 55131 Mainz, Germany; arndt@uni-mainz.de (S.A.); tenzer@uni-mainz.de (S.T.); 4 Department of Neurology, Medical Faculty, Heinrich-Heine University, 40225 Düsseldorf, Germany; eva-maria.hanschmann@med.uni-duesseldorf.de (E.-M.H.); carsten.berndt@hhu.de (C.B.)

**Keywords:** mitochondria, fusion, fission, redox, metabolism, thiol switch

## Abstract

Mitochondrial fusion and fission tailors the mitochondrial shape to changes in cellular homeostasis. Players of this process are the mitofusins, which regulate fusion of the outer mitochondrial membrane, and the fission protein DRP1. Upon specific stimuli, DRP1 translocates to the mitochondria, where it interacts with its receptors FIS1, MFF, and MID49/51. Another fission factor of clinical relevance is GDAP1. Here, we identify and discuss cysteine residues of these proteins that are conserved in phylogenetically distant organisms and which represent potential sites of posttranslational redox modifications. We reveal that worms and flies possess only a single mitofusin, which in vertebrates diverged into MFN1 and MFN2. All mitofusins contain four conserved cysteines in addition to cysteine 684 in MFN2, a site involved in mitochondrial hyperfusion. DRP1 and FIS1 are also evolutionarily conserved but only DRP1 contains four conserved cysteine residues besides cysteine 644, a specific site of nitrosylation. MFF and MID49/51 are only present in the vertebrate lineage. GDAP1 is missing in the nematode genome and contains no conserved cysteine residues. Our analysis suggests that the function of the evolutionarily oldest proteins of the mitochondrial fusion and fission machinery, the mitofusins and DRP1 but not FIS1, might be altered by redox modifications.

## 1. Introduction

The function and activity of many proteins is regulated by post-translational modifications (PTMs) of specific amino acids, including, for instance, phosphorylation of serine and tyrosine residues, ubiquitylation, and SUMOylation of lysine residues. These modifications are important for adjusting the cellular physiology to diverse challenges in order to regain homeostasis. Although cysteine is the least abundant amino acid, with 2.2% in complex eukaryotes [1], 214,000 cysteines are still encoded by the human genome [2]. Interestingly, the number of cysteines has increased throughout evolution and seems to correlate with the complexity of the organism [1].

**Figure 1:**
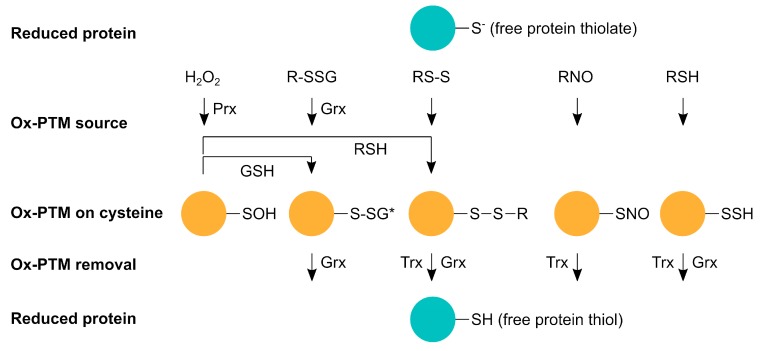
Representation scheme of reversible oxidative post-translational modifications (Ox-PTMs) on cysteines. Proteins containing free thiols or thiolates (reduced protein) exhibit reactivity towards certain oxidants, such as reactive oxygen species (H_2_O_2_), reactive nitrogen species (RNO), and reactive sulfur species (RSH), to form sulfenic acid (-SOH), S-glutathionylation (R-SSG), disulphide-bonds (RS-S), S-nitrosylation (-SNO), and S-persulfidation (-SSH). *, R-SSG and RS-S alterations represent a diverse group of modifications. All these modifications affect protein function and activity and are reversible via thioredoxins (Trx) or glutaredoxins (Grx). Under oxidative conditions, glutaredoxins are able to induce S-glutathionylation; oxidation via H_2_O_2_ is most likely facilitated by peroxiredoxins (Prx).

Cysteine is one of the most commonly post-translationally modified amino acids due to the physical availability and the acid dissociation constant pKa of its thiol (R-SH). Thiols are nucleophilic and are more reactive in their thiolate state (RS-), which can be stabilized by positively charged or protonated amino acids in their proximity [3]. In fact, cysteines can undergo at least 18 different physiological nonradical post-translational modifications [2] induced by reactive oxygen species (ROS), reactive nitrogen species (RNS), and reactive sulfur species (RSS) (Figure 1). Under physiological conditions, 5–12% of all protein cysteine residues are oxidized in cells and tissues [4]. The oxidation and reduction of cysteines that function as thiol switches, i.e., via the reversible formation of disulfide bonds or via de/glutathionylation, makes them essential for signal transduction and various cellular processes, including cell proliferation, differentiation, gene expression, and metabolism. Moreover, thiol switches exhibit not only key significance in various physiological but also pathological processes [5,6].

Hydrogen peroxide (H_2_O_2_) belongs to the group of ROS that have been studied for its important function as a second messenger in signal transduction. When H_2_O_2_ reacts with a free protein thiol, sulfenic acid (R-SOH) is formed (Figure 1), which is unstable and very reactive [7]. Sulfenic acid can be further oxidized to sulfinic (R-SO_2_H) and sulfonic acid (R-SO_3_H). Cysteine oxidation can alter protein conformation and trigger unfolding, leading to higher protein turnover, toxic aggregation, and altered cell signaling up to cell death [8].

Reactive sulfur species (RSS) and the respective thiol modification S-persulfide (-SSH) can also alter cysteines (Figure 1). Since it was first shown that H_2_S is physiologically produced and RSS has a biological function in mammals, there has been a steadily growing interest in this topic [9,10,11,12]. Next to nitric oxide (NO) and carbon monoxide (CO), H_2_S is counted amongst the gasotransmitters. However, how H_2_S, or rather downstream products, such as sulfide anion (HS-), polysulfides, and sulfhydryl radical (HS•-), act as a sulfur signal remains largely elusive. It was proposed that the canonical mitochondrial [11] and heme-dependent sulfide oxidation [13] are both important sources of RSS. Furthermore, enzyme-catalyzed transpersulfidation is crucial for the target specificity of reversible PTMs. Some results suggest that -SSH is not formed through a reaction of free thiols with RSS but rather from a nucleophilic attack from HS- with already oxidized thiols, such as disulfides, glutathionylated or nitrosylated cysteines, and sulfenic acid [14]. One hypothesis is that in this way, the reaction with excess ROS to sulfinic acid and/or RNS is hindered and the protein is hence protected from permanent damage by ROS as persulfides can be reduced. Moreover, recent data suggest that persulfidated cysteine residues can be oxidized to -SSOH, -SSO_2_H, and -SSO_3_H, which can be reduced because of the disulfide bridge [15]. H_2_S was shown to have roles in vasodilation [10], ER stress [16], and anti-apoptotic pathways [17]. For example, H_2_S regulates vasodilation through an increase of cyclic guanosine monophosphate (cGMP), potassium (K^+^) increase via opening of ATP-sensitive potassium channels (KATP), and activation of endothelial nitric oxide synthase (eNOS), which also elevates cGMP levels. The cGMP degradation by phosphodiesterase 5 (PDE5) is inhibited by H_2_S, which leads to prolonged elevated cGMP levels [18,19,20]. In addition, H_2_S relaxes vascular smooth muscle cells [21].

Reactive nitrogen species (RNS) include nitric oxide (^•^NO), nitroxyl (HNO), and peroxynitrite (ONOO^-^). They are formed from the reaction of NO with ROS in case of ONOO^-^ or H_2_S for HNO, respectively. ^•^NO itself is produced endogenously by eNOS, inducible nitric oxide synthase (iNOS), and neuronal NOS (nNOS) from L-arginine and oxygen. The cysteine PTM caused by NO is called S-nitrosylation (R-SNO) (Figure 1). The most abundant endogenously present S-nitrosothiol is S-nitrosoglutathione (GSNO), which is the major ^•^NO donor for proteins. It is formed by the transfer of a nitrosyl group (R-NO) from the heme of cytochrome *c* onto glutathione (GSH) [22]. This reaction takes place within mitochondria from which GSNO diffuses to the rest of the cell, where the –NO can be transferred to other proteins, such as the nuclear factor NF-kappa-B (NFκB), hypoxia-inducible factor 1 (HIF-1) [23], and glycerinaldehyd-3-phosphat-dehydrogenase (GAPDH) [24]. Within mitochondria, all complexes of the electron transport chain, ATP synthase, enzymes of the Krebs cycle [25,26], and β-oxidation enzymes [27] are S-nitrosylation targets. Most modified mitochondrial proteins seem to be inhibited by S-nitrosylation to restrict ROS production. However, at higher concentrations and in reaction with the superoxide radical O_2_^•-^, ^•^NO forms the more reactive ONOO^-^, leading to tyrosine nitration [28] and cellular damage [29]. GSH not only transfers NO to other proteins, it can also modify proteins itself by S-glutathionylation (R-SSG). Cysteines can spontaneously react with the glutathione disulfide (GSSG) through a nucleophilic attack of glutathione on ionized protein thiol or thiolate (Pr-S-) [30], sulfenic acid, or protein thionyl radical [31,32]. Even though S-glutathionylation has been shown to regulate protein function, for instance, through modification of transcription factors, such as the nuclear factor erythroid 2–related factor 2 (Nrf2) and NFκB [33] and mitochondrial membrane proteins [34], protein glutathionylation during oxidative distress is rather considered a protective mechanism, which is also enzymatically facilitated by glutaredoxins (see below). In general, oxidative distress describes the pathological oxidative damage of biomolecules that has been linked to various pathologies, whereas oxidative eustress describes physiological redox alterations important for the regulation of several signaling pathways and the activity of a variety of proteins [35]. For this physiological signaling function, most of the abovementioned post-translational modifications are reversible and enzymatically controlled via oxidoreductases of the thioredoxin (Trx) family (Figure 1) [36]. Oxidizing as well as reducing molecules potentially inducing post-translational modifications on their own, e.g., H_2_O_2_ or GSH, show very low reactivity with thiols. Therefore, enzymes/oxidoreductases are needed for (i) the formation and removal of these modifications and (ii) to ensure the specificity for proper signaling events [37]. Whereas Trxs are able to reduce disulfides, S-nitrosylated [38], S-persulfidated [39], and further oxidized S-persulfidated cysteine residues [15], glutaredoxins (Grxs) reduce disulfides, S-glutathionylated [40], and S-persulfidated cysteine residues [39]. Sulfenic acid cannot be reduced directly; it needs to be converted into a disulfide or a S-glutathionylation. Sulfinic and sulfonic acids are generally considered irreversible [7], although there are indications that sulfinic acid can be reversed in an ATP-dependent reaction by sulfiredoxin (Srx) [41,42]. In fact, recent data increase the number of Srx targets and indicate regulatory functions [43]. Peroxiredoxins (Prx) constitute cellular peroxidases that can reduce peroxides and transfer oxidizing equivalents to target proteins [44,45]. In addition, protein disulfide isomerases also belong to the Trx family. These enzymes are known to mediate thiol/disulfide exchange reactions, function as chaperones, and regulate protein activity [46,47,48].

## 2. The Mitochondrial Fusion and Fission Machinery

Most of the cellular reactive species are produced by mitochondria [49,50], cellular organelles with an outer (OMM) and inner (IMM) membrane that convert most of the cell’s energy to ATP by generating a proton (ΔpHm) and electrical (Δψm) gradient across the inner membrane through the respiratory chain, which drives the ATP synthase. Complex I of the respiratory chain can produce large amounts of ROS under conditions, such as a high NADH/NAD+ ratio caused by inhibition or damage to complex I [51] or reverse electron transport due to a backlog of electrons during low ATP demand [52,53], cytochrome c (Cyt c) loss, or inhibition of oxygen consumption. This leads to a relatively reduced coenzyme Q (CoQ) pool and a high proton motive force Δp [54], which then results in oxygen reduction at complex I and hence ROS production [55]. Apparently, complex I activity can also be impaired via the thiol switch of cysteine 39 of the ND3 subunit by nitric oxide, which shows a cardioprotective effect in conditions of ischemia [56]. Additionally, though still under debate and shown by in vitro conditions only, complex III of the respiratory chain has been proven as a second important ROS-producing source primarily affecting the proteins of the OMM and intermembrane space via oxidative modification, whereas the matrix proteins are rather targeted by ROS produced by complex I, indicating two separate redox-signaling pathways [57,58,59]. However, one mechanism of how ROS produced by the ETS can also enter the cytosol is via the voltage-dependent anion channel (VDAC), which resides in the outer mitochondrial membrane, thus allowing the diffusion of inner-mitochondrial superoxides into the cytosol [60]. Mitochondria also contain various members of the Trx family [36]. Trx2, and Grx1 (intermembrane space) and Grx2 (matrix) indicate functioning redox signaling [36,61,62]. In addition, mitochondria also contain two peroxiredoxins, i.e., peroxiredoxins 3 and 5 [36]

Mitochondria can form a long tubular network, which continually undergoes fusion and fission events in a regulated process termed mitochondrial dynamics necessary to adapt to environmental changes. The overall shape of the mitochondrial tubular network is determined by the balance between fusion and fission. Mitochondrial fission events also regulate and enable mitochondrial transport throughout the cells, especially throughout the neurons’ axons, while the fusion process, in contrast, can spare mitochondria from mitophagy by allowing an exchange of biolipids and mitochondrial DNA. These mitochondrial dynamics are thus vital for cellular wellbeing (reviewed in [63]).

The fusion of adjacent mitochondria can be induced by homo- or heterodimers of the GTPases mitofusin 1 and 2 (MFN1/MFN2) located at the OMM. Fusion of the IMM, in contrast, is mediated by the protein optical atrophy 1 (OPA1). Proteolytic processing of OPA1 is triggered by a loss of membrane potential and results in the induction of IMM fusion [64]. This pro-fusion mechanism seems to be controlled by cellular metabolism, as OPA1-induced fusion events are supposedly strongly linked to OXPHOS activity [64]. Fragmentation of mitochondria is primarily mediated by the cytosolic GTPase dynamin-related protein 1 (DRP1), which, after activation, translocates to the outer mitochondrial membrane, oligomerizes, and encircles the mitochondrion, thereby inducing fragmentation. Crucial for DRP1′s binding to mitochondria are other OMM proteins, including the fission 1 protein (FIS1), mitochondrial dynamics proteins of 49 and 51 kDa (MID49/51) [65], and the mitochondrial fission factor (MFF) [66]. Overexpression of the OMM protein ganglioside-induced differentiation-associated protein 1 (GDAP1), which shares homology with some glutathione-S-transferases, is closely related to mitochondrial fission, although the precise mechanism is still unclear [67,68,69].

We here hypothesize that the cysteine modification of proteins involved in mitochondrial dynamics enables cellular adaptation to altered physiological conditions that involve reactive cellular species, and review the relevant literature on this topic.

## 3. Evolutionary Conservation of Proteins Involved in Mitochondrial Dynamics

**Figure 2:**
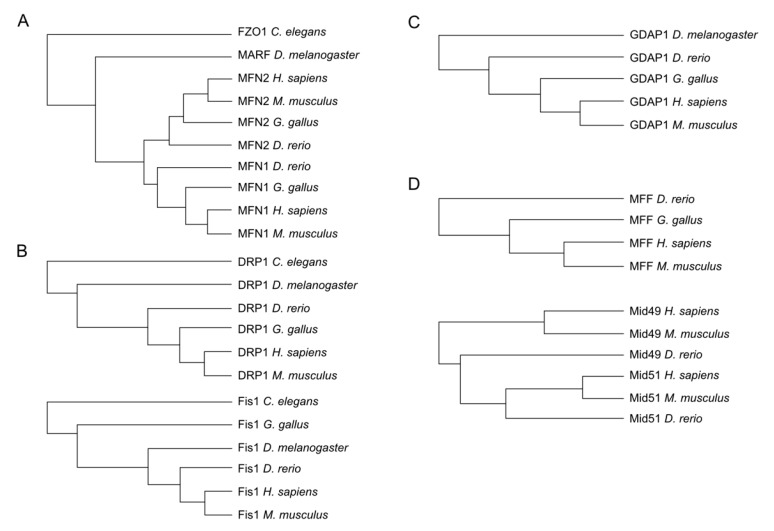
Phylogenetic trees obtained by multiple alignment of amino acid sequences from *Homo sapiens*, *Mus musculus*, *Gallus gallus*, *Danio rerio*, and *Drosophila melanogaster* when possible. The most abundant isoforms were used for the alignment. (**A**) MFN2 is present in all compared species while MFN1 was not found in *Drosophila* and *C elegans*. (**B**) DRP1 and FIS1 presented orthologs in all compared species. (**C**) GDAP1 is present in all compared species except in *C elegans*. (**D**) MFF and MID49/51 proteins were found in *Homo sapiens*, *Mus musculus*, and *Danio rerio*.

Important redox modification sites have been shown to be conserved over different taxa. Hence, to identify potential sites of the aforementioned proteins, we decided to first investigate the presence of these proteins in phylogenetically distant organisms and detect evolutionarily conserved cysteines assuming that such sites are more likely to represent sites of physiologically relevant redox modifications. Wherever possible, we obtained the protein sequence of these proteins from *Homo sapiens*, the mouse *Mus musculus*, the chicken *Gallus gallus*, the zebrafish *Danio rerio*, the fruit fly *Drosophila melanogaster*, and the nematode *Caenorhabditis elegans* using the Uniprot database (www.uniprot.org) and conducted multiple sequence alignments with Clustal Omega (www.clustal.org). Our analysis demonstrated that *C. elegans* and *D. melanogaster* possess only a single mitofusin (FZO1 and MARF1, respectively), which in vertebrates diverged into MFN1 and MFN2 (Figure 2A). DRP1 and FIS1 are also evolutionarily conserved in all these organisms (Figure 2B) while GDAP1 is also present in the fruit fly but not in the nematode genome (Figure 2C). The DRP1 receptors MFF and MID49/51 are, in contrast, evolutionarily new and only present in the vertebrate lineage (Figure 2D).

## 4. Redox Modifications of Proteins Involved in Mitochondrial Dynamics

### 4.1. Mitofusins

MFN1 and MFN2 are OMM proteins that mediate mitochondrial fusion by forming homo- or heterodimers. They are expressed most abundantly in the nervous system, as well as in skin, heart, and muscle tissue [70,71]. It has been suggested that MFN2 mediates organelle tethering between mitochondria in trans or between mitochondria and the ER while MFN1 facilitates the fusion process together with OPA1 at the inner membrane [72,73]. After GTP hydrolyzation, MFN1 apparently loses its affinity to its counterpart but has greater GTP turnover activity while MFN2 proteins still remain as dimers, making MFN2 a more effective tethering molecule [72].

**Figure 3:**
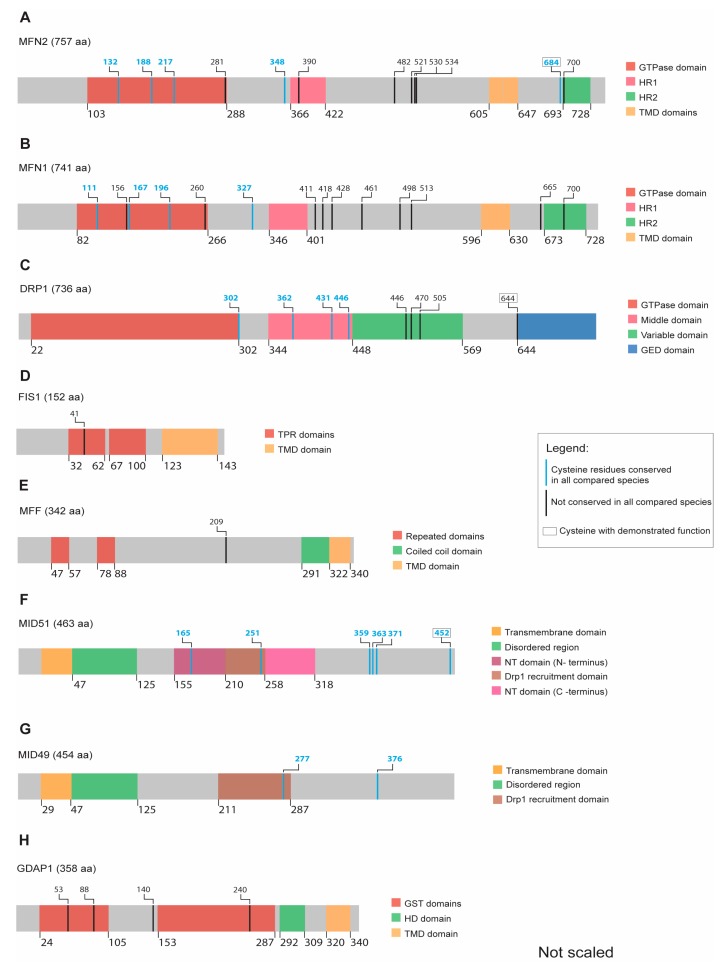
Scheme illustrating the relative position of conserved cysteines within recognizable protein domains of the indicated proteins. Colors are used to highlight protein domains, evolutionarily conserved cysteines, and cysteines with a demonstrated function. (**A**) MFN2 has five conserved cysteines out of which the C684 has a previously demonstrated redox-inducible function. (**B**) MFN1 contains four conserved cysteines but yet without any observed redox function. (**C**) DRP1 possesses four conserved cysteines, whereas a fifth not fully conserved cysteine (C644) can be redox modified. (**D**) FIS1 and (**E**) MFF do not have any conserved cysteines along their structure. (**F**) MID51 has six fully conserved cysteines, including C452, regulating the protein’s dimerization. (**G**) MID49 has two conserved cysteines (**H**) GDAP1 has no fully conserved cysteines.

MFN1 consists of 741 and MFN2 of 757 amino acids; both proteins share 82% structural similarities [74] and contain an N-terminal GTP-domain followed by a coiled-coil heptad-repeat domain (HD1) and two transmembrane domains (TMDs) separated by one amino acid (Figure 3A,B). Only MFN2 harbors a proline-rich domain between the TMD and the HD1 domain, which is most likely involved in protein–protein interactions [75,76]. The C-terminus of both mitofusins contains a second HR2 domain, which seems to be located in the intermembrane space (IMS) [77] (Figure 3A).

As mitofusins are crucial for the mitochondrial fusion process, mitofusin turnover is a highly regulated process. Mitofusins are ubiquitinated by parkin (PARK2) [78,79] during mitochondrial quality control, where PARK2 translocates to the mitochondria to orchestrate their selective elimination through mitophagy [80]. Ubiquitination of mitofusins by E3 ligases like MGRN1 and Gp78 not only controls mitochondrial turnover via mitophagy but also affects mitochondrial morphology and mitochondria-ER-contact sites (MERCS), which constitute important intracellular signaling hubs [81,82,83]. By treating primary neurons with the NO-donor A-nitrosocysteine (SNOC), Barsoum et al. observed that concomitant MFN1 expression prevented NO-induced mitochondrial fragmentation and even NO-induced cell death [84]. This may emphasize the importance of mitochondrial fusion’s ability due to mitofusin expression in order to cope with nitrosative stress. The process of mitochondrial hyperfusion, a cellular stress response induced by changes in the intracellular redox state, involves both mitofusins [85]. In line, oxidized glutathione (GSSG) addition, the core cellular stress indicator, to mitochondrial preparations stimulated mitochondrial fusion by inducing disulphide bond-mediated oligomer formation of MFN1 and cysteine 684 of MFN2 [85]. This points towards a regulation of redox-regulated mitochondrial hyperfusion into the IMS. Here, cysteines trigger dimerization of the mitofusins through disulfide bonds to drive mitochondrial fusion [77]. Accordingly, work from our laboratory demonstrated that this cysteine is involved in sensing the redox environment in the IMS [86].

In addition to the abovementioned conserved cysteine 684, both MFN1 and MFN2 contain four and five highly conserved cysteines, respectively. Three are located in the GTPase domain while the fourth one is located next to the HR1 domain in both mitofusins (Figure 3A,B and complete alignment in Supplementary Materials). The role of these evolutionarily conserved cysteines located in the cytosol remains unclear with regard to their function as potential PTMs in response to changes in the redox milieu.

### 4.2. DRP1

DRP1 is the major player in the mitochondrial fission process. The protein consists of an N-terminal GTPase and a C-terminal GTPase effector domain (GED) separated by a helical segment of amino acids [87,88]. In humans, six DRP1 isoforms are generated by alternative splicing. Isoform 1 consists of 736 amino acids and is expressed exclusively in the brain (Figure 3C). The isoforms 2 (710 amino acids) and 3 (699 amino acids) are predominantly expressed in the testis and skeletal muscle, respectively. DRP1 isoform 4 (725 amino acids) is weakly expressed in the brain, heart, and kidney. The isoform 5 (710 amino acids) occurs predominantly in the liver, heart, and kidney, while isoform 6 (749 amino acids) is expressed in neurons [89,90,91,92,93].

DRP1 exists in the cytoplasm in an equilibrium between dimeric and tetrameric forms [94]. Upon stimulation, DRP1 is recruited to mitochondria and promotes fission via interaction with its receptors FIS1, MFF, and MID49/51 located at the OMM, which allows DRP1 to form ring-like oligomers that constrict and divide the mitochondria, a process accompanied by GTP hydrolysis [95,96,97]. The ability of DRP1 to promote mitochondrial fission can be regulated by phosphorylation, ubiquitination, and SUMOylation [98].

Most importantly in the redox context, DRP1 can be regulated by S-nitrosylation of cysteine 644 (C644; position in the human protein), which is conserved from flies to humans (Figure 3C). Cho et al. showed that an NO increase provoked by amyloid-β peptide induces DRP1 S-nitrosylation at C644, leading to subsequent DRP1 dimerization and increased GTPase activity, and resulting in more fragmented mitochondria [99]. Furthermore, postmortem brain samples from Alzheimer’s disease (AD) patients presented higher levels of S-nitrosylated DRP1 when compared to control samples or samples from patients suffering from Parkinson’s disease (PD) [99]. In contrast, Bossy et al. suggested that DRP1 S-nitrosylation does not increase its GTPase activity, and that the levels of S-nitrosylated DRP1 are not changed when comparing postmortem brain samples from control, AD, and PD patients. Instead, they suggested that NO production stimulates DRP1 activity via serine 616 (S616) phosphorylation [100]. Nakamura and Lipton counterargued that Bossy et al. used recombinant DRP1 for their experiments, which was already oxidized, preventing its S-nitrosylation [101]. Additionally, it was shown that DRP1 can be trans-S-nitrosylated by cyclin-dependent kinase 5 (CDK5) in AD models [102], and that an increase in S-nitrosylated DRP1 induced by decreased S-nitrosoglutathione reductase (GSNOR) expression can occur in primary cells undergoing senescence [103]. A recent study demonstrated that the protein disulfide isomerase A1 (PDIA1) acts as a thiol reductase for DRP1. In neurodegenerative and cardiovascular diseases, PDIA1 deficiency results in increased S-nitrosylated DRP1, mitochondrial fragmentation, and endothelial senescence [104]. DRP1 phosphorylation was also associated with changes in the redox milieu. Zhou et al. showed that c-Abl-induced DRP1 phosphorylation triggers mitochondrial fragmentation upon H_2_O_2_ treatment in primary neurons [105], while Lee and Kim suggested that PDI-mediated DRP1 S-nitrosylation facilitates DRP1 S616 phosphorylation in hippocampal neurons [106]. Additionally, Tsushima et al. showed increased DRP1 S616 phosphorylation in response to lipid overload-induced oxidative stress in cardiac myocytes [107].

Besides C644, DRP1 contains four additional evolutionarily conserved cysteine residues with yet unknown functions, one at the end of the GTPase domain and three located in the middle region that connects the GTPase domain with the effector domain (Figure 3C and complete alignment in Supplementary Materials).

### 4.3. FIS1

FIS1 was the first protein shown to interact with DRP1 and to allow mitochondrial fission in the budding yeast *Saccharomyces cerevisiae* [108]. In contrast, FIS1 does not seem to be essential for mitochondrial fission in mammalian cells [109,110] and shows low tissue specificity [111]. FIS1 is a 17-kDa protein with a C-terminal OMM anchor and two cytosolic tetratricopeptide repeat (TPR) motifs (Figure 3D) [112]. A concave surface formed by the residues in the TPR motifs is believed to be the interacting region for DRP1 [113] while the N-terminal arm controls DRP1 access to the TPR motifs [114]. However, an understanding of how the position of the N-terminal arm is regulated is still lacking.

Most of the reported FIS1 regulation is associated with the regulation of its protein abundance in response to cellular stress. Glutamate-challenged HT22 cells have an upregulated FIS1 (and phosphorylated DRP1) and have a more fragmented mitochondrial shape [115]. Zhang et al. reported increased FIS1 protein levels in mice treated with 1-methyl-4-phenyl-1,2,3,6-tetrahydropyridine (MPTP; a precursor of the mitochondrial complex I inhibitor 1-methyl-4-phenylpyridinium (MPP+), a mouse model of PD, while overexpression of DJ-1 (a protein mutated in familiar PD) repressed FIS1 upregulation via RING-finger protein-5-mediated FIS1 ubiquitination and degradation [116]. In line with this, 6-hydroxydopamine (6-OHDA; another PD model) increased FIS1 (and DRP1) protein levels in PC12 cells [117], while H_2_O_2_ induced an increase in FIS1 protein levels in SH-SY5Y cells [118]. In summary, although FIS1 does not seem to be directly regulated via redox mechanisms, it seems to respond to the redox milieu via upregulation or decreased degradation. In line with this, FIS1 contains only a single cysteine, which is also not evolutionarily conserved (Figure 3D and complete alignment in Supplementary Materials). We therefore believe that a direct redox-mediated regulation of FIS1 by cysteine modification is unlikely.

### 4.4. MFF

The protein mitochondrial fission factor (MFF) was first described in a *Drosophila* cell line screen for proteins triggering mitochondrial morphology alterations. MFF knockdown caused elongated mitochondria similar to what was observed for DRP1 and FIS1 knockdown [119]. MFF contains 342 amino acids and is one of three DRP1 receptors located in the OMM facing the cytosol [119]. This protein contains two consecutive cytosolic motifs that are necessary for DRP1 recruitment [109] and a third motif of unknown function. MFF dimerization depends on a well-preserved coiled-coil domain, which precedes the C-terminal transmembrane (TM) domain (Figure 3E) [119]. MFF is broadly present in different human tissues, such as the heart, kidney, liver, brain, muscle, and stomach [119].

A PTM predictive model identified multiple serine and threonine phosphorylation sites that are potential targets for post-translational modification [120]. Mutating MFF serine 155 (S155) and S172 residues precludes mitochondrial fission promoted by AMP-activated protein kinase (AMPK) [121], a well-known intracellular energy sensor that phosphorylates multiple downstream effectors participating in cellular homeostasis [122]. These serine residues are located between the DRP1-interacting region at the N-terminus and the C-terminal mitochondrial TM domain. Additionally, S129 and S146 have been also identified as AMPK targets in hepatocytes using proteomics [123]. Interestingly, similar to MFN2, MFF has been identified as a PARK2 target for ubiquitination. Four glycine residues (G28, G88, G251, and G264) were identified within four different ubiquitin-interacting motifs [124]. Eliminating PARK2 was not sufficient to preclude MFF ubiquitination, suggesting that other proteins may play a role in MFF proteostasis [125].

MFF only contains one cysteine residue (human C209), which is located after the DRP1-interacting region but before the TM domain (Figure 3E). This residue is not conserved as it is absent in *G. gallus* (complete alignment in Supplementary Materials). In line with this, an unbiased proteomic approach, where cysteine reactivity within mitochondrial proteins was monitored by labelling cysteine-reactive residues with chemical probes followed by mass spectrometry, also failed to identify functional cysteine residues in MFF [126]. These data imply that the single and not fully conserved cysteine residue in MFF probably has no relevant function.

### 4.5. MID49/51

The MID49/51 proteins were first described in a large-scale analysis as mitochondrial proteins [127], and later redefined as novel elements of the mitochondrial fission protein group. The overexpression of MID49/51 proteins sequestered DRP1 from mitochondria and triggered an elongated mitochondrial network whereas abolishing their expression caused a fragmented mitochondrial pattern [65]. MID51 contains 463 amino acids while MID49 is slightly smaller with 454 amino acids (Figure 3F,G). MID49/51 are encoded by the mitochondrial elongation factor 1 (MIEF1) and MIEF2 genes, respectively [128].

Both proteins are located in the OMM, with most of the protein present in the cytosol to regulate DRP1-mediated mitochondrial fission. The highly conserved residues within the TMD, amino acids 26-47 of MID49, and 24-46 of MID51 are necessary for mitochondrial anchoring but are not required for interaction with DRP1 [129]. Following the TMD, an N-terminal disordered region with an absent secondary structure has been described in the crystal structure of these proteins [130]. Both proteins contain nucleotidyl transferase (NT) domains without enzymatic activity but a still preserved ADP-binding capacity for MID51 (Figure 3F,G) [130,131]. Although DRP1 recruitment by MID51 is ADP independent, ADP binding to MID51 facilitates its dimerization, which in turn stimulates DRP1 oligomerization [131]. MID49/51 also share similar α-helices and β-strands at the N-terminal position, which precedes a linker before the C-terminal containing five α helices [130]. Although both proteins share α-helices and β-sheets from the N- to C-terminus, their oligomerization domains are different [131]. While MID51 appears to be dimeric, MID49 adopts a monomeric structure. It has been proposed that MID49 weakly dimerizes and that this was not apparent in the crystal structure due to technical issues [130].

MID49/51 proteins contain DRP1 recruitment sites [132,133] and their interaction with DRP1 is independent of MFF or FIS1 [134]. The interaction of DRP1 with MID51 is GTP dependent and MID51 dimerization is necessary for mitochondrial morphology control [135]. Indeed, these differences between both MID49/51 proteins could explain different roles in mitochondrial fission.

MID49/51 proteins were not found in a proteomic screen searching for conserved functional cysteines in pure mitochondrial fractions [126]. However, MID51 can dimerize under non-reducing conditions [136]. This dimerization can be abolished by the mutation of C452 [136]. Five additional conserved cysteines distributed through the NT domain, DRP1 recruitment site, and the C-terminus were identified within MID51 (Figure 3F and complete alignment in Supplementary Materials), but no function has been defined for these until now. MID49 contains two conserved cysteine residues located within the DRP1 recruitment domain and another one close to the C-terminus (Figure 3G), which also do not have any demonstrated function so far.

### 4.6. GDAP1

GDAP1 [137] is a 41.5 kDa protein, which causes the hereditary polyneuropathy Charcot–Marie–Tooth disease 4A when mutated on both alleles [138,139]. It is anchored in the outer mitochondrial and peroxisomal membranes and mainly expressed in neurons and less in Schwann cells [140,141]. Overexpression of GDAP1 causes a more fragmented mitochondrial shape while its knockdown results in more elongated mitochondria [140]. This fission-like activity can be counterbalanced by fusion-promoting factors like mitofusins or dominant-negative DRP1 [140]. GDAP1 is anchored in the OMM with a C-terminal transmembrane domain (TMD) and faces the cytosol (Figure 3H). It has two cytosolic domains that share similarities to glutathione-S-transferases (GSTs; Figure 3H) [140]. The C-terminal hydrophobic domain (HD) of GDAP1 presumably resides in the cytosol. Truncated GDAP1 lacking the TMD has no GST-like activity [141,142], but recently, a theta-class-like GST activity was described for GDAP1. Huber et al. proposed a model where the HD domain of GDAP1 is able to regulate the GST activity of the protein. In an inactive state, the HD domain blocks the GST activity while in the active state, the amphipathic pattern of HD would trigger membrane curvature and mitochondrial fission [68]. In this model, dominant GDAP1 mutations, which cause a hereditary polyneuropathy, would adopt a super-active conformation resulting in toxic hyper-fission activity while recessive GDAP1 mutations with reduced fission activity would adopt the inactive state [68]. Furthermore, Noack et al. observed an increase of GSH in response to overexpression of wildtype but not mutant GDAP1 [143]. In line with this, *Drosophila melanogaster* studies demonstrated that not only overexpression of GDAP1 wildtype but also knockdown results in both a higher GSH concentration and higher GSH to GSSG ratio in fly thoraxes [144]. Additional studies are required to confirm the *Drosophila* observations since muscle tissue is different from neurons, where GDAP1 seems to be more important. Structurally, *Drosophila* GDAP1 shares similarities with the human GDAP1 protein, especially in respect to the GST domains. In fact, human GDAP1 overexpression was able to rescue the alterations caused by Gdap1 interference in flies [144]. This reinforces the functional similarity between the species. Niemann et al. described a disturbed GSH/GSSG balance towards increased oxidized glutathione in a mouse model with a deletion of exon 5 in the Gdap1 gene, leading to a truncated protein that lacks the TMD anchoring in the OMM [145]. These mice developed a mild hypomyelinating peripheral neuropathy, and most importantly, the lack of phenotype in the central nervous system was attributed to a compensatory function mediated by the paralogue GDAP1L1 [145], which shares a 70% sequence similarity with GDAP1 [142], including the GST domains and the C-terminal transmembrane domain [146]. GDAP1L1 expression is restricted to the brain and testes (www.proteinatlas.org) and, in contrast to GDAP1, resides in the cytosol under basal conditions but translocates to mitochondria upon increased levels of oxidized glutathione, where it is also able to induce mitochondrial fission [145].

Although all four cysteines in the human GDAP1 protein (respectively five in GDAP1L1) are only conserved in vertebrates but not in *Drosophila melanogaster* (Figure 3H and complete alignment in Supplementary Materials), potential cysteine modifications and their impact on GDAP1 function warrant further investigation. No pathogenic mutations are associated with GDAP1L1. However, its ability to translocate from the cytosol to mitochondria under altered redox conditions suggests a possible role in cellular homeostasis through redox-mediated modifications.

## 5. Conclusions

Our analysis suggests that the evolutionarily oldest proteins of the mitochondrial fusion and fission machinery, the mitofusins and DRP1 but not FIS1, are subject to redox modifications functionally relevant for mitochondrial dynamics and potentially also for mitochondrial quality control. The lack of evolutionary conservation rendered the analysis of other proteins relevant for mitochondrial dynamics more difficult. Overall, however, conserved cysteine residues are present in almost all the proteins of the mitochondrial fission/fusion machinery, offering new avenues for future investigation.

## Supplementary Materials

The following are available online at https://www.mdpi.com/2073-4409/9/4/815/s1, Protein alignments performed with CLUSTAL O (1.2.4).
