# Supplementary material for: Redox Modifications of Proteins of the Mitochondrial Fusion and Fission Machinery
Source: Cells. 2020 Mar 27;9(4):815. doi: 10.3390/cells9040815 (PMC7226787; doi:10.3390/cells9040815)
Supplement: Supplementary file 1 [file cells-09-00815-s001.pdf]

Supplementary Information

Protein alignments performed with CLUSTAL O (1.2.4).

* = fully conserved residue

: = conservation between groups of strongly similar properties

. = conservation between groups of weakly similar properties

Alignment MFN1

- Conserved cysteine residues
- GTPase domain
- HR1
- HR2
- TMD

```
MFN1 H. sapiens        ------------------------------------------------------------
MFN1 M. musculus       ------------------------------------------------------------
MFN1 G. gallus         ------------------------------------------------------------
MFN1 D. rerio          ------------------------------------------------------------
MARF D. melanogaster   MAAYLNRTISMVTGQTGPADDDRHASSTDTVDKSGPGSPLSRFNSSLQQSGSTMAANLLP 60
FZO1 C. elegans        --------------------------------MSGTAS----LVHTLPASGDSNHRGLHS 24


MFN1 H. sapiens        -----MAEPVSPLKHFVLAKKAITAIFDQLLEFVTEGSHFVEATYKNPELDRIATEDDLV 55
MFN1 M. musculus       -----MAETVSPLKHFVLAKKAITAIFGQLLEFVTEGSHFVEATYRNPELDRIASEDDLV 55
MFN1 G. gallus         ----MAEAAVSPLKHFVLAKKTITAIFEQLLDYVTEGAAFVEATYRNPELEHVATEDELA 56
MFN1 D. rerio          ----MEQLDPSPLKRFVLAKKKISEVFEQLLVYVQEGCEFVKETCANESLENIANKDQLD 56
MARF D. melanogaster   ESRLYQSNDKSPLQIFVRAKKKINDIYGEIEEYVHETTTFINALHAEAEIVD---KAERE 117
FZO1 C. elegans        LKNSRRAADNEPLLRFREAKKVLGDVYGELKDNVAELE----GVYKDIKENDFVSSEQRE 80
                                 .**  *  *** :  :: ::   * *          : .      . :

MFN1 H. sapiens        EMQGYKDKLSIIGEVLSRRHMKVAFFGRTSSGKSSVINAMLWDKVLPSGIGHITNCFLSV 115
MFN1 M. musculus       EIQGYRNKLAVIGEVLSRRHMKVAFFGRTSSGKSSVINAMLWDKVLPSGIGHTTNCFLSV 115
MFN1 G. gallus         EIQAYKKKLAVIGEVLSRRHMKVAFFGRTSSGKSSVINAMLWDKVLPSGIGHTTNCFLSV 116
MFN1 D. rerio          EIESYTSKLSIIQEVLARRHMKVAFFGRTSNGKSTVINAMLRDRVLPSGIGHTTNCFLSV 116
MARF D. melanogaster   LFESYVYKVAAIREVLQRDHMKVAFFGRTSNGKSSVINAMLREKILPSGIGHTTNCFCQV 177
FZO1 C. elegans        EIEAIGDSIKTIMDTFQRDNMKVVFFGRTSNGKSTTINAMLHEKVLPQGMGHTTCCFLQV 140
                        ::.   .:  * :.: * :***.******.***:.***** :::**.*:** * ** .*

MFN1 H. sapiens        EGTDGDKAYLMTEGSDEKKSVKTVNQLAHALHMDK----DLKAGCLVRVFWPKA----KC 167
MFN1 M. musculus       EGTDGDKAYLMTEGSDEKKSVKTVNQLAHALHMDK----DLKAGCLVHVFWPKA----KC 167
MFN1 G. gallus         EGTDGDKAYLMTEGSDEKKSVKTVNQLAHALHMDK----DLKAGCLVHVFWPKS----KC 168
MFN1 D. rerio          EGTDEDKAFLKTEGSEEEKSIKTVNQLAHALHMDE----SLDAGCLVKVFWPKT----KC 168
MARF D. melanogaster   EGSNGGEAYLMTEGSEEKLNVVNIKQLANALCQE-----KLCESSLVRIFWPRE----RC 228
FZO1 C. elegans        EGSEGEVGHLQLDDNPQKIDMKMLGKIGHALSDENSDLPAMGQDSLLKVFHPKKSESGEC 200
                       **::   ..*  :.. :: .:  : ::.:**  :      :  ..*:::* *:     .*

MFN1 H. sapiens        ALLRDDLVLVDSPGTDVTTELDSWIDKFCLDADVFVLVANSESTLMNTEKHFFHKVNERL 227
MFN1 M. musculus       ALLRDDLVLVDSPGTDVTTELDIWIDKFCLDADVFVLVANSESTLMNTEKHFFHKVNERL 227
MFN1 G. gallus         ALLRDDLVLVDSPGTDVTTELDSWIDKFCLDADVFVLVANSESTLMNTEKHFFHKVNERL 228
MFN1 D. rerio          ALLRDDLVLVDSPGTDVTTELDSWIDKFCLDADVFVLVANSESTLMNTEKHFFHKVNEKL 228
MARF D. melanogaster   SLLRDDVVFVDSPGVDVSANLDDWIDNHCLNADVFVLVLNAESTMTRAEKQFFHTVSQKL 288
FZO1 C. elegans        RLLQNDVVILDSPGVDLSPEFDSWIDKHCLDADVFVLVSNAESTLTQAEKNFFLRVAKKL 260
                        **::*:*::****.*:: ::* ***:.**:******* *:***: .:**:**  * ::*

MFN1 H. sapiens        SKPNIFILNNRWDASASEPEYMEDVRRQHMERCLHFLVEELKVVNALEAQNRIFFVSAKE 287
MFN1 M. musculus       SKPNIFILNNRWDASASEPEYMEDVRRQHMERCLHFLVEELKVVSPSEARNRIFFVSAKE 287
MFN1 G. gallus         SKPNIFILNNRWDASASEPEYMEDVRRQHMERCLTFLVDELKVIDPVEARNRIFFVSAKE 288
MFN1 D. rerio          SKPNIFVLNNRWDASAAEPEYMEDVRKQHTDRCVNFLVEELKVVDRAQAPNRIFFVSAKE 288
MARF D. melanogaster   SKPNIFILNNRWDASANEPECQESVKSQHTERCIDFLTKELKVSNEKEAAERVFFVSARE 348
FZO1 C. elegans        SKPNVFILNNRWDASAAETENIEDVKKQHLTRFRQFLVDELEVCSEREVNDRIFFVSSRE 320
                       ****:*:********* * *  *.*: **  *   **..**:* .  :. :*:****::*

MFN1 H. sapiens        VLSARKQKAQGMPESGVALAEGFHARLQEFQNFEQIFEECISQSAVKTKFEQHTIRAKQI 347
MFN1 M. musculus       VLNSRKHKAQGMPEGGGALAEGFQARLQEFQNFEQTFEECISQSAVKTKFEQHTIRAKQI 347
MFN1 G. gallus         VLSARRQKAQGMPESGGALAEGFQTRFQEFQNFEQIFEECISQSAVKTKFEQHTIRAKQI 348
MFN1 D. rerio          VLNSRMQRAQ-----GGALAEGFQDRLKEFQNFERSFEECISHSAVKTKFEQHTIRAKQI 343
```

```
MARF D. melanogaster     TLQARIEEAKGNPPHMGAIAEGFQIRYFEFQDFERKFEECISQSAVKTKFQQHSSRGKSV 408
FZO1 C. elegans          VLESRLKAR-GLVQ-KAYQAEGHGTRALEFQNFERHFEHCISRSAIHTKFEAHNRRAHEM 378
                         .*.:* .             ***.  *   ***:**: **.***:**::***: *. *.:.:

MFN1 H. sapiens           LATVKNIMDSVNLAAEDKRHYSVEEREDQIDRLDFIRNQMNLLTLDVKKKIKEVTEEVAN 407
MFN1 M. musculus          LDTVKNILDSVNVAAAEKRVYSMEEREDQIDRLDFIRNQMNLLTLDVKKKIKEVTEEVAN 407
MFN1 G. gallus            IDTVKNIMDAINVAAAEKRVLSMEEREDQKDRLDFVRNQLNILTEDTKEKIKRVTEEVEN 408
MFN1 D. rerio             TETVKDIMDQINITAAEKRVVSLEEREYLIDRLDFVRNQLNLLIQDIKKKIKAITEEVEN 403
MARF D. melanogaster      SGDMKSMLDNIYERITIFRNLKQDQKNLLTERIQGTETQMMQVTREMKMKIHNMVEEVEE 468
FZO1 C. elegans           IGKMRLNLNSVLTSAAEQRSKLQNNLNESTRTFNECRVNFTQFEKAYREQTEQLRAEVHL 438
                             ::  :: :       *    :: :     ::  . ::  .    : : . :  **

MFN1 H. sapiens           KVSCAMTDEICRLSVLVDEFCSEFHPNPDVLKIYKSELNKHIEDGMGRNLADRCTDEVNA 467
MFN1 M. musculus          KVSCAMTDEICRLSVLVDEFCSEFHPTPSVLKVYKSELNKHIEDGMGRNLADRCTNEVNA 467
MFN1 G. gallus            KVSSAMTDEICRLSVLVDEFYSDFHPSPQVLKLYKTELNKHIEDGLGKNLADRCSSEVNE 468
MFN1 D. rerio             KVSNAMAEEICRLSVIIDEFRSDFHPSPQMLKMYKSDLLSHIEQGMGKNLAFRCSDAVNA 463
MARF D. melanogaster      KVSKALNEEIWRLGVLIDEFNMPFHPERLVLNIYKKELNAHVESGLGSNLRARLSMALAM 528
FZO1 C. elegans           KVSADFFEEIARLDAIIDRFEQPFDGSSSGMTKYKEDLAIFVDKCLSSDLEARCTGGLMS 498
                         ***  :  :** **..::*.*    *.      :. ** :*  .::. :. :*   * :  :

MFN1 H. sapiens           LVLQTQQEIIENLKPLLPAGI--QDKLHTLI-PCKKFDLSYNLNYHKLCSDFQEDIVFRF 524
MFN1 M. musculus          SILQSQQEIIENLKPLLPAGI--QNKLHTLI-PCKKFDLSYDLNCHKLCSDFQEDIVFRF 524
MFN1 G. gallus            SMHQSQQEIIENLKPLLPSGA--QNQLHLLI-PCRKFDLSYDLNCHSLCADFQEDIMFHF 525
MFN1 D. rerio             SVQCSQQDMIECLKPLLPSAA--QTQLHMLI-PSRKFELSYDLNCATLCSDFQENIEFQF 520
MARF D. melanogaster      NVESAQTEMTDRMHALVPNEQLLATSTKMVV-RTQPFEMLYSLNCQNLCADFQEDLEFKF 587
FZO1 C. elegans           RIWNLENDMFQYVTKILAEPY--QNKLEEVWRYRAPFKFSICVDVPALVNDFHEDLEFRF 556
                          :   : :: : :  ::          . . :      *.:   ::   *   **:*:: *:*

MFN1 H. sapiens           SLGWSSLVHRFLGPRNAQRVLLGL--SEPIFQLPRSLASTPTAPTTP---------ATPD 573
MFN1 M. musculus          SLGWSSLVHRFLGSTNAQRVLLGL--SEPIFQVPRSLASTPTAPSNP---------AAPD 573
MFN1 G. gallus            SLGWTSLVNRFLGPKHAQRVLLGL--ADPNLQIPRPLATTPSAASLP--------AATPE 575
MFN1 D. rerio             SLGWSALVHRFLGPVNAKRALMLV---DQNLQLTAPT--TPTAPAVVQSQNSRALGQNQA 575
MARF D. melanogaster      SWGIAAMIQRFTGKVRERSKKGQPALVNRQSSIGHSV-STPTTTPVEATPVCLLPAPVVA 646
FZO1 C. elegans           TFGLHAIIRRIIAYRSGQPVTAI----NTNLLTPLSLKQQ-------SEKNSVRDAEASA 605
                         : *  :::.*: .     :          :

MFN1 H. sapiens           NASQEELMITLVTGLASVTSRTSMGIIIVGGVIWKTIGWKLLSVSLTMYGALYLYERLSW 633
MFN1 M. musculus          NAAQEELMITLITGLASLTSRTSMGIIVVGGVIWKTVGWKLISVTLSMYGALYLYERLTW 633
MFN1 G. gallus            NTSPDDFMVPLVMSLASLTSRTSMSIIVVGGVIWKTVGWKLISLSLSMYGLLYLYERLTW 635
MFN1 D. rerio             SVTQEELMLTMVNNLASVTSRTSMSVIIVGGVVWRTVGWRLIALSMSMYGLLYLYERLTW 635
MARF D. melanogaster      GITPEQLSLISRFAVSSIGSQGTVGGLVVAGVMLKTIGWRVLVGVGALYGCIYLYERLSW 706
FZO1 C. elegans           ASEEQAMMTQMVLTSAAFLANGSLGVLVVGGIVYKAVGWRVIAVGGAAYAGLYAWERMRW 665
                             : :         ::. :. ::. ::*.*:: :::**:::    : *. :* :**: *

MFN1 H. sapiens           TTHAKERAFKQQFVNYATEKLRMIVSSTSANCSHQVKQQIATTFARLCQQVDITQKQLEE 693
MFN1 M. musculus          TTRAKERAFKQQFVNYATEKLQMIVSFTSANCSHQVQQEMATTFARLCQQVDVTQKHLEE 693
MFN1 G. gallus            TTKAKERAFKQQFVNYATEKLQMIVSLTSANCSHQVQQEMATTFARLCQQVDVTQKNLEK 695
MFN1 D. rerio             TTKAKERALKKQFVDYATEKLQMIISFTSSNCSHQVQQEIASTFARLCQQVDITQKDLES 695
MARF D. melanogaster      TNSAKERTFKSQYVRHATKKLKMIVDLTSANCSHQVQQELSSTFARLCRTVDTATTDMND 766
FZO1 C. elegans           NSGAKEQHLKEQFRSHLAARMQQVSTAHTHHCETQAIREMDQVFDGLKATVGGVHREMKN 725
                         .. ***: :*.*:  : : ::: :    : :*. *. :::  .*  *    *. .  .::.

MFN1 H. sapiens           EIARLPKEIDQLEKIQNNSKLLRNKAVQLENELENFTKQFLPSSNEES- 741
MFN1 M. musculus          EIARLSKEIDQLEKIQNNSKLLRNKAVQLESELENFSKQFLHPSSGES- 741
MFN1 G. gallus            EIARLSKEIDQLENIQSHSKQLRNKAIHLENELDHFTKHFLQKSK---- 740
MFN1 D. rerio             DIQRLTDKIQKLETVQNRSKVLRHKATALEMQLEDFSSQYLRLQP---- 740
MARF D. melanogaster      ELKTLDSQLNILEANQKQLKLLRNKANYIQNELDIFEHNYISPQ----- 810
FZO1 C. elegans           DLDVQKTQIDAVDSTIRTLGTIKGKAVFLLRNLEQFASSYLRSDSPPTP 774
                         ::     ::: ::          :: **   :  :*: *   ::  .
```

Alignment MFN2

- Conserved cysteine residues
- GTPase domain
- HR1
- HR2
- TMD

```
MFN2 H. sapiens           ---------------------------------------MS-----LLFSRCNSIVTVKK 16
MFN2 M. musculus          ---------------------------------------MS-----LLFSRCNSIVTVKK 16
MFN2 G. gallus            -----------------------------VGDEAQPSRQSS-----LL---ILGFFTLKK 23
MFN2 D. rerio             ---------------------------------------MS-----LVFPRPNTSVIGKK 16
MARF D. melanogaster      MAAYLNRTISMVTGQTGPADDDRHASSTDTVDKSGPGSPLSRFNSSLQQSGSTMAANLLP 60
FZO1 C. elegans           --------------------------------MSGTAS----LVHTLPASGDSNHRGLHS 24
                                                                      *
```

```
MFN2 H. sapiens          NKRHMAEVNASPLKHFVTAKKKINGIFEQLGAYIQESATFLEDTYRNAELDPVTTEEQVL 76
MFN2 M. musculus         DKRHMAEVNASPLKHFVTAKKKINGIFEQLGAYIQESASFLEDTHRNTELDPVTTEEQVL 76
MFN2 G. gallus           DKRHMAEVNASPLKHFVTAKKKINGIFEQLAAYINESSSFLEETHKNVELDPVTTEEQVL 83
MFN2 D. rerio            DKRLMAEVNASPLKHFVTAKKKINGIFEQLGAYIKESSAFLEETYSNEELDPVTTEEQVA 76
MARF D. melanogaster     ESRLYQSNDKSPLQIFVRAKKKINDIYGEIEEYVHETTTFINALHAEAEIVDKAE---RE 117
FZO1 C. elegans          LKNSRRAADNEPLLRFREAKKVLGDVYGELKDNVAELE----GVYKDIKENDFVSSEQRE 80
                          ..     : .**  *   *** :..:: ::   : *         : : :    .

MFN2 H. sapiens          DVKGYLSKVRGISEVLARRHMKVAFFGRTSNGKSTVINAMLWDKVLPSGIGHTTNCFLRV 136
MFN2 M. musculus         DVKGYLSKVRGISEVLARRHMKVAFFGRTSNGKSTVINAMLWDKVLPSGIGHTTNCFLRV 136
MFN2 G. gallus           EVKGYLSKVSGISEVLARRHMKVAFFGRTSNGKSTVINAMLWDKVLPSGIGHTTNCFLRV 143
MFN2 D. rerio            EVRGYLSKVAGIGEVLSRRHMKVVFFGRTSNGKSSVINAMLWDKVLPSGIGHTTNCFLRV 136
MARF D. melanogaster     LFESYVYKVAAIREVLQRDHMKVAFFGRTSNGKSSVINAMLREKILPSGIGHTTNCFCQV 177
FZO1 C. elegans          EIEAIGDSIKTIMDTFQRDNMKVVFFGRTSNGKSTTINAMLHEKVLPQGMGHTTCCFLQV 140
                          ...    .:   *  :.:  *  :***.**********:.*****  :*:**.*:**** ** :*

MFN2 H. sapiens          EGTDGHEAFLLTEGSEEKRSAKTVNQLAHALHQDK----QLHAGSLVSVMWPN----SKC 188
MFN2 M. musculus         CGTDGHEAFLLTEGSEEKKSVKTVNQLAHALHQDE----QLHAGSMVSVMWPN----SKC 188
MFN2 G. gallus           EGTDGHEAFLLTEGSEEKKSVKTVNQLAHALHQDE----LLNAGSLVSVMWPN----SKC 195
MFN2 D. rerio            EGTDGNESFLLTEGSDERKSVKTVNQLAHALHQDE----DLDAGSLVCVMWPK----AKC 188
MARF D. melanogaster     EGSNGGEAYLMTEGSEEKLNVVNIKQLANALCQEK-----LCESSLVRIFWPR----ERC 228
FZO1 C. elegans          EGSEGEVGHLQLDDNPQKIDMKMLGKIGHALSDENSDLPAMGQDSLLKVFHPKKSESGEC 200
                          *::*   ..*  :.. :: .    : ::.:** :::       :  .*:: :: *.     .*

MFN2 H. sapiens          PLLKDDLVLMDSPGIDVTTELDSWIDKFCLDADVFVLVANSESTLMQTEKHFFHKVSERL 248
MFN2 M. musculus         PLLKDDLVLMDSPGIDVTTELDSWIDKFCLDADVFVLVANSESTLMQTEKQFFHKVSERL 248
MFN2 G. gallus           PLLKDDLVLMDSPGIDVTTELDSWIDKFCLDADVFVLVANSESTLMQTEKQFFHKVNERL 255
MFN2 D. rerio            ALLRDDLVLVDSPGIDVTTELDSWIDKFCLDADVFVLVANSESTLMQTEKSFFHKVNERL 248
MARF D. melanogaster     SLLRDDVVFVDSPGVDVSANLDDWIDNHCLNADVFVLVLNAESTMTRAEKQFFHTVSQKL 288
FZO1 C. elegans          RLLQNDVVILDSPGVDLSPEFDSWIDKHCLDADVFVLVSNAESTLTQAEKNFFLRVAKKL 260
                          **::*:*::****:*::  ::*.***:.**:******* *:***:  ::** **   *  ::*

MFN2 H. sapiens          SRPNIFILNNRWDASASEPEYMEEVRRQHMERCTSFLVDELGVVDRSQAGDRIFFVSAKE 308
MFN2 M. musculus         SRPNIFILNNRWDASASEPEYMEEVRRQHMERCTSFLVDELGVVDRAQAGDRIFFVSAKE 308
MFN2 G. gallus           SRPNIFILNNRWDASASEPEYMEEVRRQHMERCTSFLVDELGVVDRTQAGDRIFFVSAKE 315
MFN2 D. rerio            SSPNIFILNNRWDASANEPEYMEEVRRQHMDRCTSFLVDELRVVDRSHAGDRIFFVSAKE 308
MARF D. melanogaster     SKPNIFILNNRWDASANEPECQESVKSQHTERCIDFLTKELKVSNEKEAAERVFFVSARE 348
FZO1 C. elegans          SKPNVFILNNRWDASAAETENIEDVKKQHLTRFRQFLVDELEVCSEREVNDRIFFVSSRE 320
                         * **:*********** * *   *.*: **   *    .**..** *  .. .. :*:****::*

MFN2 H. sapiens          VLNARIQKAQGMPEGGGALAEGFQVRMFEFQNFERRFEECISQSAVKTKFEQHTVRAKQI 368
MFN2 M. musculus         VLSARVQKAQGMPEGGGALAEGFQVRMFEFQNFERQFEECISQSAVKTKFEQHTVRAKQI 368
MFN2 G. gallus           VLNARIQRAQGMPEGGGALADGFQVRMFEFQNFERRFEECISQSAVKTKFEQHTVRAKQI 375
MFN2 D. rerio            VLQARVQKAQGMPEAGGALAEGFQARMFEFQNFERRFEECISQSAVKTKFEQHTTRAKQI 368
MARF D. melanogaster     TLQARIEEAKGNPPHMGAIAEGFQIRYFEFQDFERKFEECISQSAVKTKFQQHSSRGKSV 408
FZO1 C. elegans          VLESRLKARGLVQ--KAYQAEGHGTRALEFQNFERHFEHCISRSAIHTKFEAHNRRAHEM 378
                         .*.:*::          .   *:*.   * :***:***:**.***:**::***: *. *.:.:

MFN2 H. sapiens          AEAVRLIMDSLHMAAREQQVYCEEMREERQDRLKFIDKQLELLAQDYKLRIKQITEEVER 428
MFN2 M. musculus         AEAVRLIMDSLHIAAQEQRVYCLEMREERQDRLRFIDKQLELLAQDYKLRIKQITEEVER 428
MFN2 G. gallus           AEDVRLIMDSVHVAAQEQRVYCLEMREERQERLGFIDKQLELLTQDYKRKIKQITEEVER 435
MFN2 D. rerio            SEALRLIMDSVHIAAQEQRIHCLEMKEEREDRLEFIDKQLDLLTLDCKSKIKKITEEVEK 428
MARF D. melanogaster     SGDMKSMLDNIYERITIFRNLKQDQKNLLTERIQGTETQMMQVTREMKMKIHNMVEEVEE 468
FZO1 C. elegans          IGKMRLNLNSVLTSAAEQRSKLQNNLNESTRTFNECRVNFTQFEKAYREQTEQIRAEVHL 438
                            ::  ::.:        :     :  :      :      ::  .     : : .::  **.

MFN2 H. sapiens          QVSTAMAEEIRRLSVLVDDYQMDFHPSPVVLKVYKNELHRHIEEGLGRNMSDRCSTAITN 488
MFN2 M. musculus         QVSTAMAEEIRRLSVLVDEYQMDFHPSPVVLKVYKNELHRHIEEGLGRNLSDRCSTAIAS 488
MFN2 G. gallus           QVSNAMAEEIRRLSVLVDEYQADFHPSQVVLKVYKNELHKHIEEGLGRNMSDRCSNAITA 495
MFN2 D. rerio            QVSNAMAEEIRRLAVLVDDFHMDFHPSPVVLKVYKNELHRHIEEGLGRNMSERCSSAITV 488
MARF D. melanogaster     KVSKALNEEIWRLGVLIDEFNMPFHPERLVLNIYKKELNAHVESGLGSNLRARLSMALAM 528
FZO1 C. elegans          KVSADFFEEIARLDAIIDRFEQPFDGSSSGMTKYKEDLAIFVDKCLSSDLEARCTGGLMS 498
                         :**   : *** ** .::* :.   *. .    :. **::*   .::. *. ::   * :  .:

MFN2 H. sapiens          SLQTMQQDMIDGLKPLLPVS--VRSQIDMLVPRQCFSLNYDLNCDKLCADFQEDIEFHFS 546
MFN2 M. musculus         SLQTMQQDMIDGLKPLLPVS--MRNQIDMLVPRQCFSLSYDLNCDKLCADFQEDIEFHFS 546
MFN2 G. gallus           SLQTMQQEMIDGLKPLLPLS--LRGQIDMLIPRQCFTLSYDLNCDKLCADFQEDIEFHFS 553
MFN2 D. rerio            ALQTTQTDMIDGLKPLLPIT--IREQVDKMVPRQCFALSYDLNCDKLCSDFQEDIGFHFS 546
MARF D. melanogaster     NVESAQTEMTDRMHALVPNEQLLATSTKMVVRTQPFEMLYSLNCQNLCADFQEDLEFKFS 588
FZO1 C. elegans          RIWNLENDMFQYVTKILAEPY-QNKLEEVWRYRAPFKFSICVDVPALVNDFHEDLEFRFT 557
                          : . : :* : :  ::           .        * :    ::   *   **:**: *:*:

MFN2 H. sapiens          LGWTMLVNRFLGPKNSRR-----ALMGYNDQVQR--------PIPLTPAN---PSMPPLP 590
MFN2 M. musculus         LGWTMLVNRFLGPKNSRR-----ALLGYSDQVQR--------PLPLTPAN---PSMPPLP 590
MFN2 G. gallus           LGWTMLVNRFLGPKNGRR-----ALMGYNDQIQR--------P--LTPAN---PSLPPLP 595
MFN2 D. rerio            LGWTMLVNRFLGPKNTRR-----ALMGYNDQVPR--------SLAITPVS---TSMPPFP 590
MARF D. melanogaster     WGIAAMIQRFTGKVRERSKKGQPALVNRQSSIGHSVSTPTTTPVEATPVC---LLPAPV- 644
FZO1 C. elegans          FGLHAIIRRIIAYRSGQPVTA------IN--------TNLLTPLSLKQQSEKNSVRDAEA 603
```

```
                         *   ::.*: .    :           .                 .

MFN2 H. sapiens          QGSLTQEEFMVSMVTGLASLTSRTSMGILVVGGVVWKAVGWRLIALSFGLYGLLYVYERL 650
MFN2 M. musculus         QSSLTQEELMVSMVTGLASLTSRTSMGILVVGGVVWKAVGWRLIALSFGLYGLLYVYERL 650
MFN2 G. gallus           QGSMTQEELMVSMVTGLASLTSRTSMGIIVVGGVVWKAVGWRLIALSFGLYGLLYVYERL 655
MFN2 D. rerio            QGSMTQEELMVSMVTGLASLTSRTSMGIIVVGGVIWKAVGWRLIALSVGLYGLLYVYERL 650
MARF D. melanogaster     VAGITPEQLSLISRFAVSSIGSQGTVGGLVVAGVMLKTIGWRVLVGVGALYGCIYLYERL 704
FZO1 C. elegans          SAASEEQAMMTQMVLTSAAFLANGSLGVLVVGGIVYKAVGWRVIAVGGAAYAGLYAWERM 663
                          ..   : :        ::: :. ::* :**.*:: *::***::.   . *. :* :**:

MFN2 H. sapiens          TWTTKAKERAFKRQFVEHASEKLQLVISYTGSNCSHQVQQELSGTFAHLCQQVDVTRENL 710
MFN2 M. musculus         TWTTKAKERAFKRQFVEYASEKLQLIISYTGSNCSHQVQQELSGTFAHLCQQVDITRDNL 710
MFN2 G. gallus           TWTTKAKERAFKRQFVEYAGEKLQLIVSYTGSNCSHQVQQELAGTFAQLCQQVDVTRENL 715
MFN2 D. rerio            TWTTRAKERTFKRQFVDYASEKLQLIVSYTGSNCSHQVQQELASTFSQLCQQVDVTRQQL 710
MARF D. melanogaster     SWTNSAKERTFKSQYVRHATKKLKMIVDLTSANCSHQVQQELSSTFARLCRTVDTATTDM 764
FZO1 C. elegans          RWNSGAKEQHLKEQFRSHLAARMQQVSTAHTHHCETQAIREMDQVFDGLKATVGGVHREM 723
                          *.. ***: :* *:  :   ::: :      :*. *. :*:  .*  *   *. .  ::

MFN2 H. sapiens          EQEIAAMNKKIEVLDSLQSKAKLLRNKAGWLDSELNMFTHQYLQPSR---- 757
MFN2 M. musculus         EQEIAAMNKKVEALDSLQSRAKLLRNKAGWLDSELNMFTHQYLQPSR---- 757
MFN2 G. gallus           EQEISAMNKKIEVLDSLQSKAKLLRNKAGWLDSELNMFTHQYLQQSR---- 762
MFN2 D. rerio            EDEIGDLNSKIEQLDTLQSKAKLLRNKAGWLDSELNMFIQQYLQQGR---- 757
MARF D. melanogaster     NDELKTLDSQLNILEANQKQLKLLRNKANYIQNELDIFEHNYISPQ----- 810
FZO1 C. elegans          KNDLDVQKTQIDAVDSTIRTLGTIKGKAVFLLRNLEQFASSYLRSDSPPTP 774
                         ::::   ..::: :::       ::.** ::  :*: *  .*:
```

Alignment DRP1

- GTPase domain (red)
- Middle domain (magenta)
- Variable domain (green)
- GED (blue)

```
DRP1 H. sapiens          MEALIPVINKLQDVFNTVGA--DIIQLPQIVVVGTQSSGKSSVLESLVGRDLLPRGTGIV 58
DRP1 M. musculus         MEALIPVINKLQDVFNTVGA--DIIQLPQIVVVGTQSSGKSSVLESLVGRDLLPRGTGVV 58
DRP1 G. gallus           MEALIPVINKLQDVFNTVGA--DIIQLPQIVVVGTQSSGKSSVLESLVGRDLLPRGTGVV 58
DRP1 D. rerio            MEALIPVINKLQDVFNTVGA--DIIQLPQIAVVGTQSSGKSSVLESLVGRDLLPRGTGIV 58
DRP1 D. melanogaster     MEALIPVINKLQDVFNTVGS--DSIQLPQIVVLGSQSSGKSSVIESVVGRSFLPRGTGIV 58
DRP1 C. elegans          MENLIPVVNKLQDVFATLGRKEDQIQLPQIVVVGSQSAGKSSVLENLVGRDFLPRGTGIV 60
                         ** ****:******* *:*   * ******.*:*:**:*****:*.:***.:******:*

DRP1 H. sapiens          TRRPLILQLVHVSQEDKRKTTGEENG-------------VEAEEWGKFLHTKNKLYTDFD 105
DRP1 M. musculus         TRRPLILQLVHVSPEDKRKTTGEENGKF-------QSWRVEAEEWGKFLHTKNKLYTDFD 111
DRP1 G. gallus           TRRPLILQLVHVSPEDGRKTAGDENDPATWKNPRHLSKEIDAEEWGKFLHTKNKIYTDFD 118
DRP1 D. rerio            TRRPLILQLVHVDPEDRRKTSEE--------------NGVDGEEWGKFLHTKNKIYTDFD 104
DRP1 D. melanogaster     TRRPLVLQLIYSPLDDRENRSA-EN------------GTSNAEEWGRFLHTK-KCFTDFD 104
DRP1 C. elegans          TRRPLILQLNHVALDDESKRRR-------------SNGTLLTDDWAMFEHTGSKVFTDFD 107
                         *****:*** :   :*  :                         ::*. * **  * :****

DRP1 H. sapiens          EIRQEIENETERISGNNKGVSPEPIHLKIFSPNVVNLTLVDLPGMTKVPVGDQPKDIELQ 165
DRP1 M. musculus         EIRQEIENETERISGNNKGVSPEPIHLKVFSPNVVNLTLVDLPGMTKVPVGDQPKDIELQ 171
DRP1 G. gallus           EIRQEIENETERISGNNKGISPEPIHLKIFSSNVVNLTLVDLPGMTKVPVGDQPKDIELQ 178
DRP1 D. rerio            EIRQEIENETERVSGNNKGISDEPIHLKIFSPHVVNLTLVDLPGITKVPVGDQPKDIELQ 164
DRP1 D. melanogaster     EIRKEIENETERAAGSNKGICPEPINLKIFSTHVVNLTLVDLPGITKVPVGDQPEDIEAQ 164
DRP1 C. elegans          AVRKEIEDETDRVTGVNKGISLLPISLKIYSHRVVSLSLVDLPGITKIPVGDQPVNIEEQ 167
                          :*:***:**:* :* ***:.  ** **::* .**.*:******:**:****** :** *

DRP1 H. sapiens          IRELILRFISNPNSIILAVTAANTDMATSEALKISREVDPDGRRTLAVITKLDLMDAGTD 225
DRP1 M. musculus         IRELILRFISNPNSIILAVTAANTDMATSEALKISREVDPDGRRTLAVITKLDLMDAGTD 231
DRP1 G. gallus           IRELILQFISNPNSIILAVTAANTDMATSEALKIAREVDPDGRRTLAVITKLDLMDAGTD 238
DRP1 D. rerio            IRELILKYISNPNSIILAVTAANTDMATSEALKVAREVDPDGRRTLAVVTKLDLMDAGTD 224
DRP1 D. melanogaster     IKELVLKYIENPNSIILAVTAANTDMATSEALKLAKDVDPDGRRTLAVVTKLDLMDAGTD 224
DRP1 G. gallus           IREMILLYISNPSSIILAVTPANQDFATSEPIKLAREVDAGGQRTLAVLTKLDLMDQGTD 227
                         *:*::* :*.**.******* ** *:**** :*::::** .*:*****:******* ***

DRP1 H. sapiens          AMDVLMGRVIPVKLGIIGVVNRSQLDINNKKSVTDSIRDEYAFLQKKYPSLANRNGTKYL 285
DRP1 M. musculus         AMDVLMGRVIPVKLGIIGVVNRSQLDINNKKSVTDSIRDEYAFLQKKYPSLANRNGTKYL 291
DRP1 G. gallus           AMDVLMGRVIPVKLGIIGVVNRSQLDINNKKSVADSIRDEYGFLQKKYPSLANRNGTKYL 298
DRP1 D. rerio            AMDVLMGRVIPVKLGLIGVVNRSQLDINNKKSVADSIRDEHGFLQKKYPSLANRNGTKYL 284
DRP1 D. melanogaster     AIDILCGRVIPVKLGIIGVMNRSQKDIMDQKHIDDQMKDEAAFLQRKYPTLATRNGTPYL 284
DRP1 C. elegans          AMDVLMGKVIPVKLGIIGVVNRSQQNILDNKLIVDAVKDEQSFMQKKYPTLASRNGTPYL 287
                         *:*:* *:*******:***:**** :* ::* : * ::** .*:*:***:**.**** **

DRP1 H. sapiens          ARTLNRLLMHHIRDCLPELKTRINVLAAQYQSLLNSYGEPVDDKSATLLQLITKFATEYC 345
DRP1 M. musculus         ARTLNRLLMHHIRDCLPELKTRINVLAAQYQSLLNSYGEPVDDKSATLLQLITKFATEYC 351
DRP1 G. gallus           ARTLNRLLMHHIRDCLPELKTRINVLAAQYQSLLNSYGEPVEDKSATLLQLITKFATEYC 358
```

```
DRP1 D. rerio               ARTLNRLLMHHIRDCLPELKTRINVLSAQYQSLLSSYGEPVEDMSATLLQLITKFATEYC 344
DRP1 D. melanogaster        AKTLNRLLMHHIRDCLPDLKTRVNIMATQFQSLLNSYGEDVSDKSQTLLQIITKFSSAYC 344
DRP1 C. elegans             AKRLNMLLMHHIRNCLPALKARVSIMNAQCQSDLVAFGEPVEDKNRTLLQIITRFATAYT 347
                            *: ** *******:*** **:*:.:: :* ** * ::** *.* . ****:**:*:: *

DRP1 H. sapiens             NTIEGTAKYIETSELCGGARICYIFHETFGRTLESVDPLGGLNTIDILTAIRNATGPRPA 405
DRP1 M. musculus           NTIEGTAKYIETSELCGGARICYIFHETFGRTLESVDPLGGLNTIDILTAIRNATGPRPA 411
DRP1 G. gallus             NTIEGTAKYIETSELCGGARICYIFHETFGRTLESVDPLGGLNTIDILTAIRNATGPRPA 418
DRP1 D. rerio              NTIEGTAKYIETAELCGGARICYIFHETFGRTLESVDPLGGLTTIDVLTAIRNATGPRPA 404
DRP1 D. melanogaster       CTIEGTARNIETTELCGGARMGYIFHETFGRTLDSIHPLAGLSKMDILTAIRNATGPRPA 404
DRP1 C. elegans            STIEGTARNIETTELCGGARICYIFHDTFGRSLESVNPLENLTQLDILTAIRNATGPRPA 407
                             ******: ***:******: ****:****:*:*:.** .*. :*:*************

DRP1 H. sapiens             LFVPEVSFELLVKRQIKRLEEPSLRCVELVHEEMQRIIQHCSNYSTQELLRFPKLHDAIV 465
DRP1 M. musculus           LFVPEVSFELLVKRQIKRLEEPSLRCVELVHEEMQRIIQHCSNYSTQELLRFPKLHDAIV 471
DRP1 G. gallus             LFVPEVSFELLVKRQIKRLEEPSLRCVELVHEEMQRIIQHCSNYSTQELLRFPKLHDAIV 478
DRP1 D. rerio              LFVPEVSFELLVKRQVKRLEEPSLRCVELVHEEMQRIIQHCSNYSTQELLRFPKLHDAIV 464
DRP1 D. melanogaster       LFVPEVSFELLVKRQIRRLEEPSLRCVELIHEEMQRIVQHCGNEVQQEMLRFPKLHEKIV 464
DRP1 C. elegans            LFVPEVSFELLVKRQIQRLEEPSLRCVELVHEEMQRMVQHCGFTTQQEMIRFPRLYDKIN 467
                            ***************::************:******::***.    **::***:*:: *

DRP1 H. sapiens             EVVTCLLRKRLPVTNEMVHNLVAIELAYINTKHPDFADACGLMNNNIEEQRRNRLAREL- 524
DRP1 M. musculus           EVVTCLLRKRLPVTNEMVHNLVAIELAYINTKHPDFADACGLMNNNIEEQRRNRLAREL- 530
DRP1 G. gallus             EVVTCLLRRRLPVTNEMVHNLVAIELAYINTKHPDFADACGLMNNNIEEQRRNRLAREL- 537
DRP1 D. rerio              EVVTSLLRKRLPVTNEMVHNLVAIELAYINTKHPDFADACGLMNNNIEEQRRNRM-REL- 522
DRP1 D. melanogaster       DVVTQLLRRRLPHTNVMVENIVAIELAYINTKHPDFHKDAALVPSLLKTDSDPYSQINLG 524
DRP1 C. elegans            EVVSGVLKERLKPTNELVENLVAIELAYINTKHPEFTEANLVTLLKEELLLDDRHGRSRN 527
                            :**: :*:.**  ** :*.*:*************:* .   :     :          .

DRP1 H. sapiens             --PSAVSRDKS-----SKVPSALAPASQEPSPAASAEADGKLIQDSRRETKNVASGGGGV 577
DRP1 M. musculus           --PSAGSRDKS-----SKVPSALAPASQEPPPAASAEADGKLIQDNRRETKNVPSAGGGI 583
DRP1 G. gallus             --PSSVPRDKS-----TKAPGALPPASQETVTAASAEADGKA--------------AAGA 576
DRP1 D. rerio              --PTSVPRDKM-----------------------------------------------AGG 534
DRP1 D. melanogaster       QRRANTPRNHMSPQISSHSAGSQQPQQQPPQPNSSQQQYSQVHEQ-----------NHV 573
DRP1 C. elegans            RHASTGERA-----VSAH-----GEQQLQPVPGVNGVDLNAVLQQQQQQSQNQ------- 570
                               :    *

DRP1 H. sapiens             GDGVQEPTTGNWRGMLKTSKAEELLA-E--EKSKPIPIMPASPQKGHAVNL-LDVPV-PV 632
DRP1 M. musculus           GDGGQEPTTGNWRGMLKTSKAEELLA-E--EKSKPIPIMPASPQKGHAVNL-LDVPV-PV 638
DRP1 G. gallus             GDVTQESGTGNWRGMLKSSKAEEVSA-E--EKSKPAAALPASPQKGHAVNL-LDVPV-PV 631
DRP1 D. rerio              AQAEQEGGTGTWRGMLKKGD--EGQG-E--EKTKLQSSIPASPQKGHAVNL-LDVPV-PV 587
DRP1 D. melanogaster       AENSTPSMASTWLSNILPPAPTRPDSIENSTNNTPVHNNIVSPVK--PVNLLPDVPANHN 631
DRP1 C. elegans            ------RASNGFLGLFGN----------------AAASSKTSPQEKQSANFLPEVPETQL 608
                                    :. : . :                         .** :   .*:  :**

DRP1 H. sapiens             ARKLSAREQRDCEVIERLIKSYFLIVRKNIQDSVPKAVMHFLVNHVKDTLQSELVGQLYK 692
DRP1 M. musculus           ARKLSAREQRDCEVIERLIKSYFLIVRKNIQDSVPKAVMHFLVNHVKDTLQSELVGQLYK 698
DRP1 G. gallus             ARKLSAREQRDCEVIERLIKSYFLIVRKNIQDSVPKAVMHFLVNHVKDTLQSELVGQLYK 691
DRP1 D. rerio              ARKLSAREQRDCEVIERLIKSYFLIVRKNIQDSVPKAVMHFLVNHVKDSLQSELVGQLYK 647
DRP1 D. melanogaster       PRRLTDKEQKDCDVIEHLIKSYFYIVRKSIQDSVPKAIMHFLVNYVKDNLQSELVTHLYK 691
DRP1 C. elegans            GRKLTSREQRDVAIIERLIRNYFIIVRKNIQDSVPKAIMALLVNFVRDNLQSELVRQLYK 668
                             *:*: :**:*  :**:**:.** ****.********:* :***.*:*.****** :***

DRP1 H. sapiens             SSLLDDLLTESEDMAQRRKEAADMLKALQGASQIIAEIRETHLW 736
DRP1 M. musculus           SSLLDDLLTESEDMAQRRKEAADMLKALQGASQIIAEIRETHLW 742
DRP1 G. gallus             SLLLDDLLTESEDMAQRRKEAADMLKALQRASQIIAEIRETHLW 735
DRP1 D. rerio              PALLDDLLTESEDMAQRRNEAADMLKALQKASQVIAEIRETHLW 691
DRP1 D. melanogaster       SDKAETLLNESDHIAVRRKEAADMLKALTRANHIISEIRETHMW 735
DRP1 C. elegans            PDEMDDLLAETEDMAQRRRDTLETMKALQQASVIISEVRETQVW 712
                                : ** *::.:* **.:: : :***  *. :*:*:***::*
```

Alignment Fis1

- TPR motifs (red)
- TMD (yellow)

```
Fis1 H. sapiens             ----------------------------MEAVLNELVSVEDLLKFEKKFQSEK-AAGSVS 31
Fis1 M. musculus            ----------------------------MEAVLNELVSVEDLKNFERKFQSEQ-AAGSVS 31
Fis1 G. gallus              RPIRARSAPQRPIAPHSTPSCPIAPHSAPQRPISPCRPRFPPQALERRYAEES-HRGAPS 59
Fis1 D. rerio               ----------------------------MEAVVSDIVAPEDLKKFEKKYNAEL-VKGPVS 31
Fis1 D. melanogaster        ----------------------------MEDLLNEVVPQEDLERFEKKYHHELELDGEVT 32
Fis1 C. elegans             --------------------------MDYGTILEERTNPAVLMNAREQYMRQC-ARGDPS 33
                                                              :.            ..::  :    *  :

Fis1 H. sapiens             KSTQFEYAWCLVRSKYNDDIRKGIVLLEELLPKGS-KEEQRDYVFYLAVGNYRLKEYEKA 90
```

```
Fis1 M. musculus         KSTQFEYAWCLVRSKYNEDIRRGIVLLEELLPKGS-KEEQRDYVFYLAVGNYRLKEYEKA 90
Fis1 G. gallus           RRCRFEYAWGLLRSRYNSDVRKGVAMFRELMADGA-PEEQRDCVYYLAVGNYRLKEYEQA 118
Fis1 D. rerio            RDTTFEYAWCLIRSKYTNDIVKGIQLLEELVHTSK-KDDQRDFLFYLAVANYRLKEYERA 90
Fis1 D. melanogaster     TDTKFEYAFCLVRSRYTNDVRKGIMILEELARTH-H-PDGRRDYIYYLAFGNARIKEYTSG 90
Fis1 C. elegans          AASTFAFAHAMIGSKNKLDVKEGIVCLEKLLRDDEDRTSKRNYVYYLAVAHARIKQYDLA 93
                           * :*  :: *: . *: .*:  :.:*        :*: ::***..: *:*:*  .

Fis1 H. sapiens          LKYVRGLLQTEPQNNQAKELERLIDKAMKKDGLVGMAIVGGMALGVAGLAGLIGLAVSKS 150
Fis1 M. musculus         LKYVRGLLQTEPQNNQAKELERLIDKAMKKDGLVGMAIVGGMALGVAGLAGLIGLAVSKS 150
Fis1 G. gallus           LKYVGALLQAEPGNTQGLRLQQLVRACMHRGEPHCAP---------------------- 155
Fis1 D. rerio            LKYIRTLLKNEPDNKQALELEKLIKDALKKDGLVGMAIVGGIGLGVAGLAGLIGLAVSKG 150
Fis1 D. melanogaster     LKYCRAFLDIES-NDQVRSLEEYIKKEIDKEVAKGMVVAGGAALVLGGILGL-GIAMARN 148
Fis1 C. elegans          LGYIDVLLDAEGDNQQAKTLKESIKSAMTHDGLIGAAIVGGGALALAGLVAIFSMSRK-- 151
                        * *   :*. *  * *   *:. :   : :

Fis1 H. sapiens          KS---- 152
Fis1 M. musculus         KS---- 152
Fis1 G. gallus           ------
Fis1 D. rerio            AKS--- 153
Fis1 D. melanogaster     KQKREK 154
Fis1 C. elegans          ------
```

Alignment MFF

Repeated domains
Coiled coil domain
TMD

```
MFF H. sapiens          MSKGTSSDTSLGRVSRAAFPSPTAAEMAEISRIQYEMEYTEGISQRMRVPEKLKVAPPNA 60
MFF M. musculus         --------------------------MAEISRIQYEMEYTEGISQRMRVPEKLKVAPPNA 34
MFF G. gallus           -------------MSGAAFPSP-AAEMAEINRLQYEMEYTEGISQRMRVPEKLKVAPQNA 46
MFF D. rerio            --------------------------MAEVNRIHYELEYTEGISQQMRIPERLKIASGSS 34
                                                  ***:.*::**:********:**:**:**:*   .:

MFF H. sapiens          DLEQGFQEGVPNASVIMQVPERIVVAGNNEDVSFSRPADLDLIQSTPFKPLALKTPPRVL 120
MFF M. musculus         DLEQEFQDGVPNASVIMQVPERIVVTGNNEDISFSRPADLDLIQSTPFKPLALKTPPRVL 94
MFF G. gallus           DLEKNIPEGFPNASVTMQVPERIVVAGNSEDIPLSRPPDLDLLQSTPFKPLALKTPPRVI 106
MFF D. rerio            EEPPGLLNA--SHSTMMLVPERIVIAGDDNDARFGRPRDLDLIQSTPLETVELKTPPRVL 92
                        :    : :.   . *. * ******::*:.:*  :.** ****:****:: : *******:

MFF H. sapiens          TLSERPLDFLDLERPPT-TPQN-EEIRAVGRLKRERSMSEN-AVRQNGQLVRNDSLWHRS 177
MFF M. musculus         TLSERPLDFLDLERPLP-TPQS-EESRAVGRLKRERSMSEN-AVRQNGQLVRNDSI---- 147
MFF G. gallus           SLSDRPLDFLDLEKPPQQTPQN-EEVRSVGRLKRERSMSEN-ATRQNGQLARNDSM---- 160
MFF D. rerio            TLNDQPLDFLEPEPAANSTAQPREEMKSHFRSRREQCRSENSTMRRNGQINKHDF----- 147
                        :*.::*****: *      * *  ** ::  * :**:. *** : *:***: ::*

MFF H. sapiens          DSAPRNKISRFQAPISAPEYTVTPSPQQARVCPPHMLPEDGANLSSARGILSLIQSSTRR 237
MFF M. musculus         ---------------------VTPSPPQARVCPPHMLPEDGANLSSARGILSLIQSSTRR 186
MFF G. gallus           ------------------------------------------------------------
MFF D. rerio            -------------------ASPSPSRAPVRVCPPLISPEDSQNLNSASGVLNYIKSTTRR 188

MFF H. sapiens          AYQQILDVLDENRRPVLRGGSA-AATSNPHHDNVRYGISNIDTTIEGTSDDLTVVDAASL 296
MFF M. musculus         AYQQILDVLDENRRPVLRGGSA-AATSNPHHDNVRYGISNLDAAIEGASDDMTVVDAASL 245
MFF G. gallus           --------------PVLRGGSAATTSSNPHHDNTRYGLSNMDTTLEGTPDDMTVVDAASL 206
MFF D. rerio            AYQQVLEVLDDSQRG-------------------RASLVTFDASVENTPDDAGLTDAASL 229
                                                          *  .:  .:*:::*.: **  :.*****

MFF H. sapiens          RRQIIKLNRRLQLLEEENKERAKREMVMYSITVAFWLLNSWLWFRR 342
MFF M. musculus         RRQIIKLNRRLQLLEEENKERAKREMVMYSITVAFWLLNSWLWFRR 291
MFF G. gallus           RRQIIKLNRRLQLLEEENKERAKREMIMYSITVAFWLLNSWLWFRR 252
MFF D. rerio            RRQIIKLNRRLQLLEHENKERAKREMVMYSLTVAFWLVNSWIWLRR 275
                        ***************.**********:***:******:***:*:**
```

Alignment GDAP1

GST domains
HD
TMD

```
GDAP1 H. sapiens        -----MA--ERQEEQ-------RGSPPLRAEGKADAEVKLILYHWTHSFSSQKVRLVIAE 46
GDAP1 M. musculus       -----MA--RRQDEA-------RAGVPLRVEGPPDKEVHLILYHWTHSFSSQKVRLVIAE 46
GDAP1 G. gallus         -----------------------------MAGPGGAAAPLVLYHWTQSFSSQKVRLAIAE 31
GDAP1 D. rerio          MAVENIGEGESQDEKEILIKKDAQDSGEIVESTKTKASKLILYHWTQSFSSQKVRLAIAE 60
```

```
GDAP1 D. melanogaster   -----MS--EQTKEIEALP-----PTLQDFKAPDLPANKPVLFFHPYNFHAQKVLMVFYE 48
                                                    .        :*:.   .* :*** :.: *

GDAP1 H. sapiens        KALKCEEHDVSLPLSEHNEPWFMRLNSTGEVPVLIHGENIICEATQIIDYLEQTFLDERT 106
GDAP1 M. musculus       KALKCEEHDVSLPLSEHNEPWFMRLNSAGEVPVLVHGENIICEATQIIDYLEQTFLDERT 106
GDAP1 G. gallus         KALTCEEHDVNLPLSEHNEPWFMRLNASGEVPVLIHGENIICEATQIIDYLEATFVDAEV 91
GDAP1 D. rerio          KGLQCEDYDVSLPLSEHNEPWFMRLNPTGEVPVLVHDNHVICDPTQIMDYLEQNFCDEQT 120
GDAP1 D. melanogaster   KKIDFFPYVVDLCNGEQYSNWFLNLNPKGDVPVLQDGALVIPSSTHIINYVESKFRGDRS 108
                        * :    : *.*  .*: . **:.**  *:****  ..   :* . *:*::*:* .* . .

GDAP1 H. sapiens        PRLMPDKESMYYPRVQHYRELLDSLPMDAYTHGCILHPELTVDSMIPAYATTRIRSQIGN 166
GDAP1 M. musculus       PRLMPDEGSMYYPRVQHYRELLDSLPMDAYTHGCILHPELTVDSMIPAYATTRIRSQIGN 166
GDAP1 G. gallus         PRLMPEEGSMYYPRVQHYRELLDSLPMDAYTHGCILHPELTVDSMIPAYATSRIRSQISN 151
GDAP1 D. rerio          PKLIPEEGSTYYHRVQHYRELLDSLQMDAYTHGCILHPEITVDSHIPAYATTHIRTQIGN 180
GDAP1 D. melanogaster   --LKPAHNSKEFDQMLIFEQAMARLPVGTLSLGSFIHDDLKLVPKAPFIGPVRQ-SCLKN 165
                          * *  . *   : ::   :.: :   *  :.: : *.::* ::.:    *   .   :  : : *

GDAP1 H. sapiens        TESELKKLAEENPDLQEAYIAKQKRLKSKLLDHDNVKYLKKILDELEKVLDQVETELQRR 226
GDAP1 M. musculus       TESELKKLAEENPDLQEAYIAKQKRLKSKLLDHDNVKYLKKILDELEKVLDQVETELQRR 226
GDAP1 G. gallus         TESELKKLAEENPDLQDAYIAKQKRLKSKLLDHDNIKYLKKILDELEKVLDQVETELQRR 211
GDAP1 D. rerio          TESELKKLAVENPDLKDAYIAKQRRLKSKLFDHDNMKYLKKLLDELENVLDQVETELQRR 240
GDAP1 D. melanogaster   NEKVLDLLRHSVDEQATKKAALQHKLDIQLRRH----ELASSREEFQKVLDAVRHFLLYV 221
                        .*. *. *   .   :      * *::*. :*   *      *  .   :*:::*** *.   *

GDAP1 H. sapiens        NEETPEEGQ-QPWLCGESFTLADVSLAVTLHRLKFLGFARRNWGNGKRPNLETYYERVLK 285
GDAP1 M. musculus       NEETPEEGN-QPWLCGESFTLADVSLAVTLHRLKFLGFARRNWGHGKRPNLETYYERVLK 285
GDAP1 G. gallus         NEETPEDGS-QPWLCGEFFSLADVSLAVTLHRLKFLGLARRNWGNGKRPNLEAYYERVLK 270
GDAP1 D. rerio          SEETPEEGSQQAWLCGDFFSIADVSLAVTLHRLKFLGLSRRYWGNGMRVNLETYYERVLD 300
GDAP1 D. melanogaster   EQELTAQAPRVEWLTGDELSVADISLGLLLHRLYQLGFENQFWTFGKLPQVEAYFLRFRQ 281
                        .:*   :.    ** *: :::**:**.: ****  **: .: *  *    ::*:*: *. .

GDAP1 H. sapiens        RKTFNKVLGHVNNILISAVLPTAFRVAKKRAPKVLGTTLVVGLLAGVGYFAFMLFRKRLG 345
GDAP1 M. musculus       RKTFNKVLGHVNNILISAVLPTAFRVAKKRAPKVLGSTLVVGLLVGMGYFAFMLFRRRLG 345
GDAP1 G. gallus         RKAFHKVLGHVNNILISAVLPTAFRVAKKRAPRVLGTTLMVGMLAGMAYFALVCLRKRFA 330
GDAP1 D. rerio          RETFRRVLGHVNNILISAVLPTAFRVAKKKAPAFVGATLLVGLIGGATYLAFIYFKKRLL 360
GDAP1 D. melanogaster   RESFHRLQPSNFAILREMW---------TRTPGNYK------LGAGAGFLGMAMFAAFAH 326
                        * :*.::      ** .             .::*            :  *  ::.:  :

GDAP1 H. sapiens        SMILAFRPRPNYF 358
GDAP1 M. musculus       SMILALRPRPNYF 358
GDAP1 G. gallus         NMMLSIRTRQNYF 343
GDAP1 D. rerio          VS----------- 362
GDAP1 D. melanogaster   K------------ 327
```

Alignment GDAP1L1

Conserved cysteine residues

GST domains

```
GDAP1L1 H. sapiens      MATPNNLTPTNCSWWPISALESDAAKPAEAPDAPE--AASPAHWPRESLVLYHWTQSFSS 58
GDAP1L1 M. musculus     MATPNNLTPTNCSWWPISALESDAAKPVETPDAPE--ASSPAHWPKESLVLYHWTQSFSS 58
GDAP1L1 G. gallus       MATPNNVTPTNCSWWPISALENDAGKSKEGEENQD--PTDPALRSQDRLVLYHWTQSFSS 58
GDAP1L1 D. rerio        MASSNNVTPTNCSWWPISAMEEDGKIPD-GEESHEPTIHEHKPYSKDRLVLYHWTQSFSS 59
                        **: **:*************:*.*.        :  :     .      :: ************

GDAP1L1 H. sapiens      QK---VRLVIAEKGLVCEERDVSLPQSEHKEPWFMRLNLGEEVPVIIHRDNIISDYDQII 115
GDAP1L1 M. musculus     QKRLQVRLVIAEKGLACEERDVSLPQSEHKEPWFMRLNLGEEVPVIIHRDNIISDYDQII 118
GDAP1L1 G. gallus       QK---VRLVIAEKGLPCEERDVSMPLLEHKEPWFMRLNLGEEVPVIIHRDNIISDYNQII 115
GDAP1L1 D. rerio        QK---VRLVINEKGLLCEERDVSLPLTEQKEPWFMRLNLGEEVPVFIHGDTIVSDYNQII 116
                        **   ***** **** *******:*  *:****************:** *.*:***:***

GDAP1L1 H. sapiens      DYVERTFTGEHVVALMPEVGSLQHARVLQYRELLDALPMDAYTHGCILHPELTTDSMIPK 175
GDAP1L1 M. musculus     DYVERTFTGEHVVALMPEAGSPQHARVLQYRELLDALPMDAYTHGCILHPELTTDSMIPK 178
GDAP1L1 G. gallus       DYMERNFTGENVPQLIPEPGTLLHSRVLQYRELLDSLPMDAYTHGCILHPELTTDSMIPK 175
GDAP1L1 D. rerio        DYIETNFVGDTVAQLIPDEGTPMYARVQQYRELLDGLPMDAYTHGCILHPELTTDSMIPK 176
                        **:* .*.*: *  *:*: *:  ::** *******.**************************

GDAP1L1 H. sapiens      YATAEIRRHLANATTDLMKLDHEEEPQLSEPYLSKQKKLMAKILEHDDVSYLKKILGELA 235
GDAP1L1 M. musculus     YATAEIRRHLANATTDLMKLDHEEEPQLSEPYLSKQKKLMAKILEHDDVSYLKKILGELA 238
GDAP1L1 G. gallus       YATAEIRRHLANASTELMKLDHEEEPPLTEPYLSKQKKLMAKILEHDNVNYLKKILGELG 235
GDAP1L1 D. rerio        YATAEIRRHLANAASELMKLDHE-EPQLTEPYLSKQKKLMAKILDHDNVNYLKKILGELA 235
                        *************:::******* ** *:**************:**:*.*********.

GDAP1L1 H. sapiens      MVLDQIEAELEKRKLENEGQKCELWLCGCAFTLADVLLGATLHRLKFLGLSKKYWEDGSR 295
GDAP1L1 M. musculus     MVLDQIEAELEKRKLENEGQTCELWLCGCAFTLADVLLGATLHRLKFLGLSKKYWEDGSR 298
```

```
GDAP1L1 G. gallus     MVLDQIEAELEKRKLEYQGQKCELWLCGCVFTLADILLGATLHRLKFLGLSKKYWEDGSR 295
GDAP1L1 D. rerio      MVLDQVEAELEKRKLEYQGQKCELWLCGPTFTLADICLGATLHRLKFLGLSRKYWEDGSR 295
                      *****:********** :**.******* .*****: **************:********

GDAP1L1 H. sapiens    PNLQSFFERVQRRFAFRKVLGDIHTTLLSAVIPNAFRLVKRKPPSFFGASFLMGSLGGMG 355
GDAP1L1 M. musculus   PNLQSFFERVQRRFAFRKVLGDIHTTLLSAVIPNAFRLVKRKPPSFFGASFLMGSLGGMG 358
GDAP1L1 G. gallus     PNLQSFFDRIQKRFAFRKVLGDIHTTLLSAVVPNAFRLVKRKPPSFFGASFLMGSLGGMG 355
GDAP1L1 D. rerio      PNLQSFFERVQKRYAFRKVLGDIHTTLLSAVLPNAFRMVKKKPPSFFGASFLMGSLGGMG 355
                      *******:*:*:*:*****************:*****:**:*******************

GDAP1L1 H. sapiens    YFAYWYLKKKYI 367
GDAP1L1 M. musculus   YFAYWYLKKKYI 370
GDAP1L1 G. gallus     YFAYWYLKKKYI 367
GDAP1L1 D. rerio      YFAYWFLKKKYM 367
                      *****:*****:
```

Alignment Mid49

Transmembrane region
Disordered region
Drp1 recruitment domain

```
Mid49 H. sapiens      MAEFSQKRGKRRSDEGLGSMVDFLLANARLVLGVGGAAVLGIATLAVKRFIDRATSPRDE 60
Mid49 M. musculus     MAEFSQKQRKQSGSEGLGSVVDFLLANARLVLGVGGAAVLGIATLAVKRLIDRATSPPDE 60
Mid49 D. rerio        ----MYYSGKRRGEDGIAAVIDFLLSNARLVLGVGGAAVLGIATLAVKRLIERAGRPPDE 56
                               *: ..:*:.:::****:***********************:*:**  * **

Mid49 H. sapiens      DD--TKADSWKELSLLKATPHLQPRPPPA-------------ALSQPVLPLAPSSSAPEG 105
Mid49 M. musculus     DD--TKGDSWKELSLLRATSPQKPQPPPA-------------AFSQPLATGSPSPSVPVE 105
Mid49 D. rerio        EPDKKMTDSWEELSLVSASPKLLHKGIEGVVMKQIAAATKKAELSQPIP----MPSSEPQ 112
                      :   .  ***:****: *:     :   .             :***:       *

Mid49 H. sapiens N    PAETDPEVTPQLSSPAPLCLTLQERLLAFERDRVTIPAAQVALAKQLAGDIALELQAYFR 165
Mid49 M. musculus     PTPIHSPTTPKFSTIAPLCLTFQERLLAFERKHVITPEAHVTLAKQLAGDIALELQAYLR 165
Mid49 D. rerio        RCDADPPPPQRRKRMDLCVLTFADRLQQYYRTRVCLSAEEVCVAQQRALDIATEIQAFLH 172
                          .     : .      **: :**  : * :*     .* :*:* * *** *:**:::

Mid49 H. sapiens      SKFPELPFGAFVPGGPLYDGLQAGAADHVRLLVPLVLEPGLWSLVPGVDTVARDPRCWAV 225
Mid49 M. musculus     SKFPELPFGALVPGGPLYDGLQAGTAEHVRLLAPLELEPGLWSLVPGVDTVAREPRCWAV 225
Mid49 D. rerio        SKHPDMPLGDMTLAGSLLDDLQVVTADHACLIVPLQLESSLWTPIAGEDTFLGHPQYCMV 232
                      **.*::*:* :. .* * *.**. :*:*. *:.** ** .**: : * **.  .*:   *

Mid49 H. sapiens      RRTQLEFCPRGSSPWDRFLVGGYLSSRVLLELLRKALAASVNWPAIGSLLGCLIRPSMAS 285
Mid49 M. musculus     RRTQLEFHPRGCSPWDRFLVGGYLSSRVLLELLRKALSASVNWPAIGSLLGCLIWPDVAS 285
Mid49 D. rerio        RRENLEYFPRGRSYWDRHLLGAYLSSRLVCEQLSKAVMESMNWPSLTGTLECEVRPVLGS 292
                      ** :**: *** * ***.*:*.*****:: * * **:  *:***:: . * * : * :.*

Mid49 H. sapiens      EELLLEVQHERLELT----VAVLVAVPGVDA-DDRLLLAWPLEGLAGNLWLQDLYPVEAA 340
Mid49 M. musculus     EELLLKVQHECLEFT----LAVLMVVPGAST-DDRLLLAWPLEGLASNLWLQDLYPVETA 340
Mid49 D. rerio        PELKLEIQGLTQSSHDSGERFYITVLPTVRLGQMTLMAQREITGSFDYVWYQSLYSSETA 352
                       ** *::*    .          : .:* .  :  *:    : *  . :* *.**  *:*

Mid49 H. sapiens      RLRALDDHDAGTRRRLLLLLCAVCRGCSALGQLGRGHLTQVVLRLGEDNVDWTEEALGER 400
Mid49 M. musculus     RLRALDDQDAGTRRRLLLLLCGICRGHPALVRLGWSHLTQVVLHLGEEEVAWTEEALGER 400
Mid49 D. rerio        RLAALDQADSGARRKCLKTLKAVCRNCPALRKLNGSHISNVVLHMSEKETDWSESAFSNR 412
                      ** ***: *:*:**: *  * .:**. ** :*.  .*:::***::.*.:. *:*.*:.:*

Mid49 H. sapiens      FLQALELLIGSLEQASLPCHFNPSVNLFSSLREEEIDDIGYALYSGLQEPEGLL- 454
Mid49 M. musculus     FLQALEFLVGSLEQASLPCHFNPSVNLLGNFREEEIDDIGYVLYSGLQVPESLF- 454
Mid49 D. rerio        FLQAITELIGYLETGVLPSYFKANVNLFQGFTEDDIDEMGFMLYCAVTEPEILLI 467
                      ****:  *:* ** . **.:*: .***: .: *::**::*: **..:  ** *:
```

Alignment MiD51

Transmembrane region
Disordered region
NT domain (N-terminus)
Drp1 recruitment domain
NT domain (C-terminus)

```
Mid51 H. sapiens      -MAGAGERKGKKDDNGIGTAIDFVLSNARLVLGVGGAAMLGIATLAVKRMYDRAISAPTS 59
Mid51 M. musculus     -MAGAGERKGKKDDNGIGTAIDFVLSNARLVLGVGGAAMLGIATLAVKRMYDRAISAPTS 59
Mid51 D. rerio        MAGVNGDRKGKKDDNGLGTAIDFVLSNAKLVLGVGGAAMLGIATLAVKRMYDRALSAPSS 60
                        .  *:*********:***********:*************************:***:*
```

```
Mid51 H. sapiens      PTRLSHSGKRSWEEPNWMGS-PRLLNRDMKTGLSRSLQTLPTDSSTFDTDTFCPPRPKPV 118
Mid51 M. musculus     PTRLSHSGKRSWEEPNWMGS-PRLLNKDMKAGLSRSLQTLPTDSSAFDTDTFCPPRPKPL 118
Mid51 D. rerio        PTKADPSGRRSWEEPSWLGSSPRTLNHDMKQNVSRSLQTLPTSSSSFKPDSLHRGLA-RG 119
                      **: . **:******.*:** ** **:*** .:*********.**:*. *::

MiD51 H. sapiens      ARKGQVDLKKSRLRMSLQEKLLTYYRNRAAIPAGEQARAKQAAVDICAELRSFLRAKLPD 178
MiD51 M. musculus     ARRGQVDLKKSRLRMSLQEKLLSYYRNRAAIPAGEQARAKQAAVDICAELRSFLRAKLPD 178
MiD51 D. rerio        GRPAKAELQRARLRLSLQEHLWAFFHERVTIPSEEQSVARRAALDICAELRVFLHAKLPD 179
                      .* .:.:*:::***:****:* :::::*.:**: **: *::**:******* **:*****

Mid51 H. sapiens      MPLRDMYLSGSLYDDLQVVTADHIQLIVPLVLEQNLWSCIPGEDTIMNVPGFFLVRRENP 238
Mid51 M. musculus     MPLRDMYLSGSLYDDLQVVTADHIQLIVPLVLEQNLWSCIPGEDTIMNVPGFFLVRRENP 238
Mid51 D. rerio        MPLREMYLSGSLYDDLQVVTADHAQLMVPLILEKNLWSSIPGEDTIMNIPGFSLVRRENL 239
                      ****:****************** **:***:**:****.*********:*** ******

Mid51 H. sapiens      EYFPRGSSYWDRCVVGGYLSPKTVADTFEKVVAGSINWPAIGSLLDYVIRPAPPPEALTL 298
Mid51 M. musculus     EYFPRGSSYWDRCVVGGYLSPKTVADTFEKVVAGSINWPAIGSLLDYVIRPAPPPEALTL 298
Mid51 D. rerio        EYFPRGSSYWDRCMVGGYLSPKSVLEVFEKLVAGSINWPAIGSVLDYVIRPVVPSETLTL 299
                      *************:*********:* :.***:************:*******. * *:***

Mid51 H. sapiens      EVQYERDKHLFIDFLPSVTLGDTV-LVAKPHRLA--QYDNLWRLSLRPAETARLRALDQA 355
Mid51 M. musculus     EVQYEKDKHLVIDFLPSVTLGDTV-LVARPHRLA--QYDNLWRLSLRPAETARLRALDQA 355
Mid51 D. rerio        EVQYETDRRLYVDFLPLLVMEDGVSLIAKPHRLAAERHENLWRQSFRVAETAKLRALDQE 359
                      ***** *::* :**** :.: * * *:*:*****  :::**** *:* ****:******

Mid51 H. sapiens      DSGCRSLCLKILKAICKSTPALGHLTASQLTNVILHLAQEEADWSPDMLADRFLQALRGL 415
Mid51 M. musculus     DSGCRSLCLKILKAICKSTPALGHLTASQLTNVILHLAQEEADWSPDMLADRFLQALRGL 415
Mid51 D. rerio        DAGCRSVCLKILKAVCKLNPALARLNASQLTNAILLLSEQEGDWTQEALADRFMQLLRAL 419
                      *:****:*******:** .***.:*.******.** *:::*.**: : *****:* **.*

Mid51 H. sapiens      ISYLEAGVLPSALNPKVNLFAELTPEEIDELGYTLYCSLSEPEVLLQT- 463
Mid51 M. musculus     ISYLEAGVLPSALNPKVNLFAELTPQEIDELGYTLYCSLSEPEVLLQT- 463
Mid51 D. rerio        VGHLEAGRMPCTLNLKVNLLCELTPQEIDELGYTLYCALSDPESLLRTV 468
                      :.:**** :*.:** ****:.****:***********:**:** **:*
```
